# Neural signatures of social inferences predict the number of real-life social contacts and autism severity

**DOI:** 10.1038/s41467-023-40078-3

**Published:** 2023-07-20

**Authors:** Anita Tusche, Robert P. Spunt, Lynn K. Paul, Julian M. Tyszka, Ralph Adolphs

**Affiliations:** 1 Division of the Humanities and Social Sciences, California Institute of Technology, Pasadena, CA 91125 USA; 2 Department of Psychology, Queen’s University, Kingston, Ontario, K7L 3N6 Canada; 3 Division of Biology and Biological Engineering, California Institute of Technology, Pasadena, CA 91125 USA

**Keywords:** Social neuroscience, Social behaviour, Human behaviour

## Abstract

We regularly infer other people’s thoughts and feelings from observing their actions, but how this ability contributes to successful social behavior and interactions remains unknown. We show that neural activation patterns during social inferences obtained in the laboratory predict the number of social contacts in the real world, as measured by the social network index, in three neurotypical samples (total *n* = 126) and one sample of autistic adults (*n* = 23). We also show that brain patterns during social inference generalize across individuals in these groups. Cross-validated associations between brain activations and social inference localize selectively to the right posterior superior temporal sulcus and were specific for social, but not nonsocial, inference. Activation within this same brain region also predicts autism-like trait scores from questionnaires and autism symptom severity. Thus, neural activations produced while thinking about other people’s mental states predict variance in multiple indices of social functioning in the real world.

## Abstract (web summary)

How our ability to infer the cognitive and emotional states of other people manifests in both neural activity and real-world social behavior is not fully understood. Here, the authors show neural activation patterns in the right posterior superior temporal sulcus during a social inference task predict the number of social contacts in both neurotypical and autism groups.

## Introduction

Although it is a platitude that we are a social species, the number of—and participation in—social relationships differs dramatically across individuals. This variance in people’s social ties and network characteristics can impact health and well-being^1–4^. The possibility that some of the variability in people’s social networks might have a neural basis has received considerable attention. A popular evolutionary account, the social brain hypothesis, proposed that the large size of the primate neocortex evolved in order to sustain the demands of increasingly complex social interactions and larger social groups^5,6^. While this hypothesis is debated^7–9^, it has motivated a number of neuroimaging studies that attempt to link individual differences in social network characteristics to variance in brain structure, specifically in brain areas implicated in processing social information and behaviors^10,11^. For example, online social network size on social media has been shown to correlate with variability in gray matter density in the right superior temporal sulcus (STS), a region implicated in social perception and processing biological motion^12^ (but see ref. ^13^). Moreover, the size and complexity of social networks have been reported to correlate with the volume of the amygdala, a structure associated with processing facial expressions^12,14,15^ (also see ref. ^16^ for lesion evidence). Evidence for the link between variance in social network size and the brain’s gray matter volume spans primate species^5,17–19^.

A smaller number of neuroimaging studies in humans and non-human primates have started to examine how these findings based on brain structure might also extend to brain function, specifically in brain regions associated with social cognition and social information processing. For instance, initial correlational evidence linked variability in people’s social network characteristics to brain activation in the amygdala or posterior superior temporal sulcus (pSTS) in response to social stimuli such as biological motion^20^ or social working memory^14,21^. Similarly, measures of functional connectivity between brain regions have been correlated with social network variability, both during social exclusion^22^ and task-free rest periods^23–26^, and social network size of active interactions on social media (Twitter)^27^.

Previous associations of variance in brain structure and function with social network characteristics motivate the hypothesis that neural correlates of people’s abilities to process social information could serve as predictive markers for one particular social network metric: the commonly used social network index (SNI)^4,10,15,26,28^, which captures the number of social contacts. However, prior work leaves open critical limitations with respect to generalizability and specificity and with respect to delineating a possible cognitive process that might explain the association found. First, all prior work has been exclusively based on conventional correlations; no prior study has used a predictive framework with cross-validation, leaving out-of-sample generalizability unclear. Here we address this issue using both cross-validation and two replication samples of neurotypical individuals. Second, no prior studies have further tested generalizability by incorporating participant samples whose social cognition and social behavior are outside the neurotypical range. Here we address this issue by testing a fourth group with autism. By using a predictive model trained on data from neurotypical individuals and tested on data from the autism group, we provide the most direct test of generalization to populations that are neurodiverse. Third, prior studies have typically been unable to link social network size to a well-isolated, specific cognitive process. Here we used a well-validated neuroimaging task that isolates social inferences, both to define brain regions of interest (ROIs) as the basis for all subsequent analyses, and for the multivariate activation patterns that served as input features for our prediction of individuals’ number of social contacts. To probe the (social) specificity of our findings, we further incorporated a control task using nonsocial (factual) inferences. These features of our approach provide the most compelling and specific evidence to date that social network characteristics like the number of social contacts can be predicted from a specific neural marker: regional patterns of brain activity produced when people think about others’ mental states.

The ability to infer other people’s mental states is a pervasive component of social cognition: we attribute others’ beliefs, intentions, emotions, or desires all the time, in the real world, as in films and novels. This social inference ability is sometimes broadly referred to as “theory of mind” or “mentalizing”^29–31^. The capacity to infer other people’s minds is a fundamental component of social intelligence^32^, is thought to be required for successful social interactions^33^, contributes to prosocial behaviors^34,35^, and has been linked to variance in social network characteristics^11,22,36^. Typically, this social ability develops between 3–5 years of age (^37,38^, for a review, see ref. ^30^), but is notably delayed in children with autism spectrum disorder (ASD)^39^. Moreover, deficits in the ability to make social inferences have been suggested to contribute to the diagnostic difficulties in communication and social interaction in ASD (for a review, see ref. ^39^). Further evidence that the ability to make social inferences is related to the capacity for successful social interactions comes from behavioral findings in typically developed adults: variability in people’s ability to make social inferences covaried with the size of their support clique, an index of an individual’s ability to successfully build and maintain a social network^40^.

**Fig. 1:**
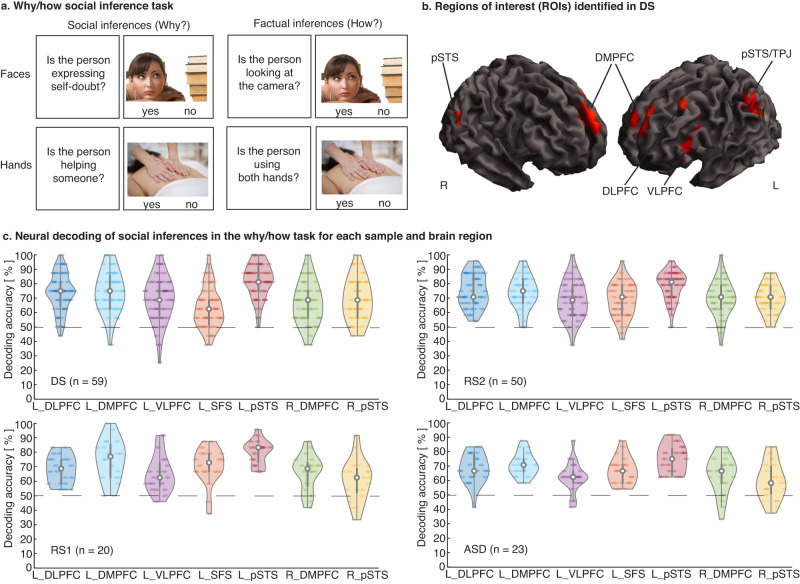
Measuring social inference processing in the brain. **a** Why/how social inference task (fMRI). In each task block, participants respond to yes/no questions that require either social inferences about other people’s internal states (why? high level of inference; left column) or factual inferences (how? low level of inference; right column). The task uses two stimulus sets (emotional facial expressions, top row; intentional hand actions, bottom row)^48^. An additional condition using nonsocial stimuli to elicit why/how inferences is not displayed (implemented for the replication samples 1 and 2 (RS1, RS2) and the autism spectrum disorder (ASD) group). **b** Brain regions from which social inference could be decoded in the neurotypical discovery sample (DS; why vs. how; *p* < 0.05, FWE whole-brain corrected at the voxel level; simple *t* test of whole-brain decoding accuracy maps at the group level implemented in SPM12; see Table 2 for details and full names of abbreviated brain areas). These clusters served as regions of interest (ROIs) for the neural prediction of real-world social behavior in all four participant samples. **c** Activation patterns in the ROIs (identified in the DS group) also decoded participants’ engagement in social inferences (why vs. how) in RS1, RS2, and ASD groups, verifying the ROIs selected based on results in DS. For each ROI and participant sample, decoding accuracies are shown for each individual (circles within violin plots; *n* biologically independent samples: DS = 59, RS1 = 20, RS2 = 50, ASD = 23). Dotted lines illustrate the chance level of the prediction (50%). Central white marks of boxplots (gray bars within violin plots) indicate ROI-based median accuracies, which were well above chance in each ROI and sample (all *p*’s < 0.05, permutation tests, FDR-corrected). Edges of boxplots indicate the 25th–75th percentiles and whiskers of boxplots illustrate minima and maxima. Decoding accuracies of social inferences were comparable across ASD and the matched RS1 groups (two-sample *t* tests, *p*’s > 0.52, FDR corrected). Source data are provided as a Source Data file, for exact *p* values see Table S8.

As reviewed above, there are thus associations between the number of social contacts and neural variability, and between the number of social contacts and social cognition. What is missing is a direct link between all three: can we predict the number of social contacts from the specific brain activations produced while participants engage in a specific social inference task? Here we focused on social inference both because of its plausible link to social relationships and because this cognitive process is known to engage a specific set of brain structures. The brain network includes the pSTS, temporo-parietal junction (TPJ), temporal poles, and medial prefrontal cortex (mPFC)^41–46^. While the specifics can vary depending on the details of the social inference task^44^, this evidence suggests that we can capture social inference processing from activation in a set of well-described brain areas. One of the best-validated tasks for social inference processing in fMRI studies is the why/how task (Fig. 1a). This task has strong psychometric properties and reliably activates the network of brain regions described above, even in single participants^47,48^.

We used the why/how task together with questionnaire- and observation-based assessments of social behavior to address three specific questions: First, can we predict the real-world number of social contacts based on the multivariate pattern of neural activations to social inferences measured in the laboratory? Second, does this activation pattern during social inferences generalize to predicting other indices of social behavior, such as autism-like traits? Third, does this prediction generalize to people with autism? To address these questions, we applied multivariate prediction techniques^49,50^ to brain activations obtained during the why/how task^47,48^. Going beyond conventional correlation approaches, we used a predictive framework with cross-validation to quantify whether the pattern of brain activations during social inference is associated with the number of social contacts and other indices of real-world social behavior. Finally, we also tested the specificity of the findings by comparing them to predictions obtained when using an fMRI task with an identical structure but requiring causal inferences about nonsocial events rather than social ones. We tested these questions in four participant samples: a neurotypical DS, two neurotypical replication samples (RS1, RS2), and a sample of high-functioning people with ASD (see Table 1 for details; all statistical evaluations of neural predictions used non-parametric permutation tests).

**Table 1:** Demographic information and summary scores of social functioning

	Discovery sample (DS)	Replication sample 1 (RS1)	Replication sample 2 (RS2)	Autism spectrum disorder sample (ASD)
Sample size (total sample)	59 (60)	20 (20)	50 (55)	23 (25)
Age (M ± SD)	28.29 ± 5.21 [19,40]	28.45 ± 6.51 [21,46]	33.56 ± 7.28 [21,49]	27.52 ± 8.25 [18,48]
Sex (male/female)	33/26	13/6	31/19	17/6
IQ scores (WASI-II)				
Full-scale IQ	108.14 ± 10.14 [90,132]	111.35 ± 9.20 [97,130]	105.92 ± 7.41 [93,127]	107.70 ± 15.40 [77,133]
Verbal comprehension index	108.91 ± 11 [87,135]	110.50 ± 12.32 [85,137]	107.48 ± 6.08 [97,120]	107.43 ± 16.53 [74,135]
Perceptual reasoning index	105.14 ± 10.94 [83,128]	110.15 ± 7.92 [87,121]	102.58 ± 9.93 [84,128]	107.04 ± 13.02 [74,127]
Social network metrics				
Network size	16.68 ± 10.23 [3,57]	21.35 ± 13.88 [6,57]	13.42 ± 8.79 [0, 37]	13.96 ± 7.60 [4,31]
Network diversity	5.07 ± 1.63 [2,9]	4.94 ± 1.75 [1,9]	4.34 ± 1.66 [0, 8]	4.87 ± 1.79 [2,8]
Embedded networks	1.68 ± 1.59 [0, 7]	3.00 ± 2.29 [0, 8]	1.24 ± 1.15 [0, 4]	1.26 ± 1.14 [0, 4]
Autism quotient scores (AQ)	15.55 ± 2.73 [8,20]	13.35 ± 4.53 [6,22]	-	-
Social responsiveness scale (SRS-2)	-	-	50.44 ± 8.38 [38,67]	-
Symptom severity scores in ASD				
ADOS SA	-	-	-	7.52 ± 1.50 [5,10]
ADOS RRB	-	-	-	6.86 ± 2.51 [0, 10]

Mean ± standard deviation [range], *IQ* intelligence quotient, *WASI-II* Wechsler Abbreviated Scales of Intelligence-II, *ADOS* autism diagnostic observation schedule scores, *ADOS* SA symptom severity in the social affect domain, *ADOS* *RRB* restricted, repetitive behavior domain, *AQ* Baron–Cohen Autism Quotient. Some entries are derived from incomplete data. Source data are provided as a Source Data file.

## Results

### Behavior

**Fig. 2:**
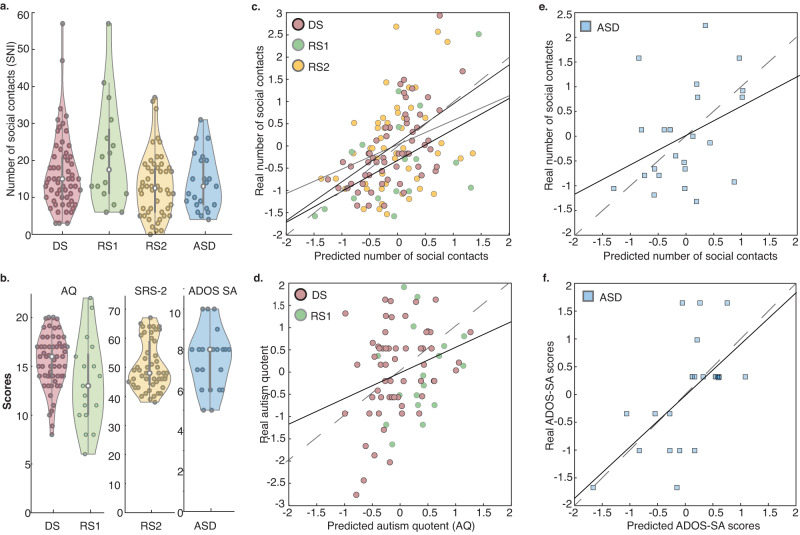
Neural prediction of real-world social functioning. **a** Violin plots of the number of real-life social contacts (SNI scores of social network size) in the neurotypical Discovery Sample (DS, *n* = 59), neurotypical Replication Samples 1 and 2 (RS1, *n* = 17; RS2, *n* = 50), and Autism Spectrum Disorder group (ASD, *n* = 23). **b** Violin plots of autism-like traits in neurotypical samples (Autism Quotient, AQ in DS [*n* = 59] and RS1 [*n* = 17]; Social Responsiveness Scale-2 in SRS-2, *n* = 50) and autism symptom severity in the social affective domain in the ASD group (ADOS SA, *n* = 21). Circles within violin plots represent participants; gray bars indicate the 25th–75th percentiles; box plot whiskers illustrate the minima and maxima; white dots indicate the median. **c** Illustration of the match of predicted and actual number of real-life social contacts (SNI, *z* scored) from brain activation in the right pSTS (DS: *r* = 0.46 [−0.38, 0.26], *R*^2^ = 0.21, *p* = 0.004; RS1: *r* = 0.52 [−0.49, 0.48], *R*^2^ = 0.27, *p* = 0.036; RS2: *r* = 0.30 [−0.39, 0.28], *R*^2^ = 0.09, *p* = 0.043; Pearson correlations, *p* values derived from non-parametric permutation tests; leave-one-participant-out-cross-validation). Solid lines indicate trend lines for each sample; dashed lines are for reference and indicate the theoretical perfect correspondence of values on both axes (predicted = real scores). **d** Neural prediction of autism-like trait scores (Autism Quotient, AQ, *z* scored for comparability across behavioral measures) in the community samples (DS, RS1) from activation patterns in the right pSTS (*r* = 0.29 [−0.34, 0.24], *R*^2^ = 0.09, *p* = 0.021, Pearson correlation, permutation test). **e** Neural activation patterns the pSTS from all three neurotypical samples predict the number of social contacts (SNI scores, *r* = 0.38 [−0.35, 0.35], *R*^2^ = 0.14, *p* = 0.037) and **f** symptom severity (ADOS SA scores) in the ASD group (*r* = 0.60 [−0.36, 0.36], *R*^2^ = 0.36, *p* = 0.001; Pearson correlations, permutation tests; cross-sample prediction: train on data of all neurotypical individuals, test on data of the ASD group). Higher ADOS SA scores indicate more severe symptoms in the social affective domain. Source data are provided as a Source Data file.

Number of social contacts: the number of social contacts in real life was assessed by the SNI (http://www.midss.org/content/social-network-index-sni) and varied considerably across individuals in all four participant samples: a neurotypical discovery sample (DS), two neurotypical replication samples (RS1, RS2), and a sample of high-functioning people with ASD (Table 1). For completeness, Table 1 also reports scores of network diversity and the number of embedded networks of the SNI. While the current study focused on the number of social contacts, high positive correlations among SNI measures (Table S1) indicate that they might capture related metrics of social network characteristics. Importantly, SNI scores showed sufficient variance across participants in each sample to justify the neural prediction of individual differences (Fig. 2a).

Autism-like traits and autism symptom severity: Table 1 also displays summary scores for three additional measures of social functioning that we investigated in different participant samples: in neurotypical individuals, we measured autism-like traits with the Autism Quotient (AQ, in DS and RS1)^51^ and the Social Responsiveness Scale-2 (SRS-2, Adult Form, Self-Report, in RS2)^52^. Both the AQ and SRS-2 have self-report versions used to screen for ASD in adults. We report the SRS-2 for our larger replication sample RS2 as this measure has been suggested to be preferable to the AQ for assessing the Broad Autism Phenotype^53^. Within the general population, autism-like traits have been proposed to be continuously distributed^52^. Consistent with this notion, the distribution and range of behavioral scores of social functioning (AQ, SRS-2) in all three neurotypical subject samples allowed us to justify neural decoding of individual differences (Fig. 2b). In the ASD group, we measured autism symptom severity from the calibrated severity scores of the ADOS (social affect domain [ADOS SA]; restricted, repetitive behavior domain [ADOS RRB]^54,55^). We focus on symptom severity in the social affective domain (ADOS SA). As for the SNI scores, ADOS SA scores indicated a sufficient variance for analyses of individual differences in autism severity in this sample (Fig. 2b).

Behavior on the why/how task (fMRI): consistent with previous implementations of the why/how task^47,48^, participants in all samples performed at (or close to) ceiling (average response accuracy [%]: DS = 94.61 ± 2.42, RS1 = 94.95 ± 2.82, RS2 = 94.80 ± 2.45, ASD = 93.04 ± 4.11; M ± SD; see Table S2 for summary scores on response accuracies, RTs, and d’s separately for each task condition and sample). Accuracy scores in the ASD group were significantly lower compared to those in the two non-matched neurotypical samples (DS: *t*(80) = 2.14, [0.11, 3.03], *p* = 0.04; RS2: *t*(71) = 2.28, [0.22, 3.30], *p* = 0.03; independent sample *t* tests, two-tailed, uncorrected), but not significantly different from accuracies in the matched RS1 group (*t*(41) = 1.75 [−0.30, 4.12], *p* = 0.09). As expected, accuracy scores for neurotypical community samples were comparable (DS vs. RS1: *t*(77) = 0.52, [−0.96, 1.64], *p* = 0.60; DS vs. RS2: *t*(107) = 0.42, [−0.73, 1.12], *p* = 0.68; RS1 vs. RS2: *t*(68) = 0.22, [−1.20, 1.50], *p* = 0.83). Consistently high response accuracies (Table S2) demonstrate that participants in all four participant samples gave valid task performances.

### fMRI results: outline of the analysis approach

Our analysis strategy proceeded in several steps: First, we used an established why/how fMRI task^47,48^ to locate functionally delineated regions of interest (ROIs) from which social inference processing could be decoded in a discovery group of neurotypical individuals (DS, *n* = 59). Second, we examined whether neural activation patterns from these ROIs predict individual differences in people’s number of social contacts in DS. Third, using the same set of ROIs for consistency across all participant groups, we replicated this prediction of individual differences in two community samples of neurotypical individuals (replication samples 1 and 2, RS1 and RS2). Fourth, we ensured the specificity of our findings to social inferences by comparison with neural activations obtained during nonsocial causal inferences. Fifth, we investigated the generalizability of our findings to other measures of peoples’ social functioning outside of the lab: autism-like traits in neurotypical individuals (AQ scores^51^ in DS and RS1, and SRS-2^52^ scores in RS2). Finally, we extended our investigation to individuals diagnosed with Autism Spectrum Disorder (ASD). We examined whether activation patterns subserving social inferences in neurotypical individuals would generalize to participant populations that are neurodiverse. Specifically, we tested if they predict individual differences in the number of social contacts and symptom severity in the social domain (ADOS SA^54^) in the ASD group.

### Identifying brain regions from which social inference can be decoded in neurotypical adults (why/how fMRI task)

First, we localized activation patterns in the brain from which social inferences can be decoded in the DS group (‘why’ vs. ‘how’ inferences in the why/how fMRI task, see Fig. 1a). Using multivariate decoding together with a searchlight approach^35,49,50,56^, we found that activation patterns in the bilateral dorsomedial prefrontal cortex (DMPFC), bilateral pSTS, left dorsal and ventral lateral prefrontal cortex (DLPFC, VLPFC), and left superior frontal sulcus (SFS) reliably decoded social inferences in the why/how task (*p* < 0.05, FWE corrected at the voxel level for the whole brain as implemented in SPM12). See Table 2 for details and Fig. 1b for an illustration. These brain regions identified in the DS group served as regions of interest (ROIs) for the subsequent cross-validated prediction of the number of social contacts in real life (and other behavioral measures of interest) in all four participant samples. Thus, ROIs were independently defined concerning our key analyses—the neural prediction of individual differences in peoples’ social functioning outside of the lab—and different participant samples (RS1, RS2, and ASD), reducing the risk of producing false positive results and circular analysis (i.e., double dipping)^57^.

**Table 2:** Brain regions decoding social inferences in the why/how task in the DS group (whole-brain searchlight decoding)

Brain region	Side	*k*	*t*		MNI	
				*x*	*y*	*z*
pSTS (posterior superior temporal sulcus)/ TPJ (temporo-parietal junction)/ SMG (supramarginal gyrus)	L	754	19.09	−46	−66	24
pSTS	R	80	8.93	48	−62	22
DMPFC (dorsomedial prefrontal cortex)	L	430	10.31	−8	60	26
DMPFC	R	161	7.60	4	58	24
SFS (superior frontal sulcus)	L	34	9.68	−30	20	50
DLPFC (dorsal lateral prefrontal cortex)	L	232	8.99	−46	12	36
VLPFC (ventral lateral prefrontal cortex)	L	84	6.42	−50	26	14

Results are reported at a statistical threshold of *p* < 0.05, FWE corrected at the voxel level for the whole brain (cluster threshold of five voxels); only peak activations of clusters are reported; *L* left hemisphere, *R* right hemisphere, *k* cluster size in voxels, *MNI* Montreal Neurological Institute. Source data available on OSF (10.17605/OSF.IO/RNT8S).

Complementary univariate analyses further confirmed the involvement of the identified brain regions during social inference processing (Table S6), mirroring findings of previous implementations of the why/how task^47,48^. Figure S1 illustrates the overlap between univariate and multivariate results. We defined ROIs based on the results of the multivariate decoding analysis as they have been suggested to be more sensitive than the univariate results^58^, and we intended to closely match the multivariate analysis approach for the key prediction of social network scores.

### Neural prediction of individual differences in the number of social contacts in DS

Having localized brain areas that reliably decode social inference processing in the DS group (Table 2; Fig. 1b), we proceeded to the key question of the study: do neural activation patterns obtained during social inferences predict individual differences in the number of social contacts? We tested this hypothesis using an ROI-based cross-participant prediction approach for SNI scores^56^. Multivoxel activation patterns in the right pSTS reliably predicted differences in the number of social contacts across individuals in the DS (*r* = 0.46 [−0.38, 0.26], *R*^2^ = 0.21, *p* = 0.004, permutation test, significant at *p*_adj_ < 0.05 FDR corrected^59^; for illustration see Fig. 2c). No other ROI yielded predictions above the chance level (Table 3).

**Table 3:** Neural prediction of social network size scores in the DS group

Region of Interest (ROI)	Accuracy [5th, 95th]	*R*^2^	*P* values
R_pSTS	0.46 * [−0.38, 0.26]	0.21	0.004 *
L_pSTS	−0.03 [−0.29, 0.30]	0.0008	0.524
L_DMPFC	−0.13 [−0.31, 0.39]	0.02	0.709
R_DMPFC	0.16 [−0.34, 0.26]	0.03	0.167
L_SFS	0.16 [−0.38, 0.25]	0.03	0.123
L_DLPFC	−0.11 [−0.33, 0.28]	0.01	0.681
L_VLPFC	−0.11 [−0.36, 0.30]	0.01	0.658

Prediction effect sizes (accuracy scores) are represented by Pearson correlation coefficients of predicted and self-reported numbers of real-life social contacts (supplemental *R* square values are also reported, error degrees of freedom = 57). Upper and lower boundaries of prediction accuracies achieved by chance (empirical null distributions) are displayed for each ROI [5th and 95th percentile]. Permutation tests assessed the statistical significance of the ROI-wise prediction accuracies (*P* values). * indicates *p* values that survive FDR correction (*p*-_adj_ = 0.028)^59^, controlling for the false discovery rate across multiple ROI-wise tests. *L_/R_* left/right hemisphere. ROIs available on OSF (10.17605/OSF.IO/RNT8S). *R_pSTS/L_pSTS* right and left posterior superior temporal sulcus, *L_DMPFC/R_DMPFC* right and left dorsomedial prefrontal cortex, *L_SFS* left superior frontal sulcus, *L_DLPFC* left dorsal lateral prefrontal cortex, *L_VLPFC* left ventral lateral prefrontal cortex.

### Prediction of individual differences in the number of social contacts in two replication samples (RS1 and RS2)

We showed that neural activation patterns for social inferences predict variance in the number of social contacts in a community sample of DS (cross-validated, localized to the right pSTS). To further validate this finding, we aimed to replicate our results in two datasets of neurotypical individuals: a smaller replication sample RS1 (*N* = 20) matching the ASD group (see below) and a larger replication sample RS2 (*N* = 50).

First, as a sanity check, we validated the ROIs. Specifically, we confirmed that neural activation patterns in the same ROIs derived from the DS sample (Table 2) decoded social inferences in the why/how task also in RS1 and RS2. For every ROI, we carried out a decoding analysis of social inferences (‘why’ vs. ‘how’) (separately for RS1 and RS2). These analyses yielded decoding accuracies well above chance level (50%) for each ROI and participant sample (all *p*’s <0.05, permutation tests, FDR corrected^59^). For details, see Table 4; for an illustration, see Fig. 1c. The results demonstrate that all ROIs (identified in DS) reliably decode social inferences across all three neurotypical participant samples (and ASD, see below).

**Table 4:** ROI-wise decoding of social inference in the why/how task in each participant sample

Regions of interest (ROI)	Decoding accuracy [%]			
	Discovery sample (DS)	Replication sample 1 (RS1)	Replication sample (RS2)	Autism spectrum disorder sample (ASD)
L_pSTS	82 [31,69]	80 [36,65]	79 [36,64]	75 [38,62]
R_pSTS	69 [39,61]	61 [44,56]	70 [40,60]	60 [45,55]
L_DMPFC	74 [35,65]	75 [38,62]	74 [38,62]	71 [40,60]
R_DMPFC	66 [40,60]	66 [42,58]	70 [40,60]	64 [43,57]
L_SFS	63 [42,58]	71 [40,61]	69 [40,59]	66 [42,58]
L_DLPFC	74 [36,64]	68 [41,59]	75 [37,63]	68 [41,59]
L_VLPFC	69 [39,61]	65 [42,58]	69 [40,59]	63 [44,57]

All analyses pertain to the fixed set of regions of interest (ROIs) obtained from the DS group. Thus, ROIs are completely independent of RS1, RS2, and ASD. ROI-wise decoding results for DS are displayed for comparative purposes only. Values represent the average percentage of cross-validated correct classification (decoding accuracy) of social inferences (why vs. how) based on ROI-wise activation patterns in the why/how task in each sample (columns) and ROI (rows). Values in brackets represent the 5th and 95th percentile of the empirical null distributions of decoding accuracies achieved by chance (1000 folds). Neural activation patterns in each ROI and participant sample predicted social inferences well above chance (permutation tests, all *p*’s <0.05, FDR corrected), validating the ROI selection across samples. Source data are provided as a Source Data file. *R_pSTS/L_pSTS* right and left posterior superior temporal sulcus, *L_DMPFC/R_DMPFC* right and left dorsomedial prefrontal cortex, *L_SFS* left superior frontal sulcus, *L_DLPFC* left dorsal lateral prefrontal cortex, *L_VLPFC* left ventral lateral prefrontal cortex.

Second, we aimed to replicate our key finding from the DS group: neural activation patterns for social inference in the right pSTS predict variance in people’s number of social contacts. Matching results in DS, neural response patterns in the right pSTS reliably predicted SNI scores in RS1 (*r* = 0.52 [−0.49, 0.48], *R*^2^ = 0.27, *p* = 0.036) and RS2 (*r* = 0.30 [−0.39, 0.28], *R*^2^ = 0.09, *p* = 0.043, permutation tests; Fig. 2c). For completeness, supplemental analyses confirmed that predictive information was specific to activation patterns in the pSTS and did not extend to other ROIs listed in Table 2 (all *p*’s > 0.14, FDR corrected). In sum, we successfully replicated the key result: neural activation patterns in pSTS during social inferences predict individual differences in people’s real-world number of social contacts across three neurotypical participant samples.

Supplemental analyses tested whether variance in the ability to decode social inferences in the why/how task across participants might be linked to variance in SNI scores. To this end, for each ROI, we correlated participants’ ROI-wise decoding accuracies in the why/how task with their SNI scores (separately for each participant sample). We found that participant-specific decoding accuracies for social inferences per se (see Fig. 1c for illustration) were not correlated with their SNI scores (neither in DS, RS1, or RS2; all *p*’s > 0.31, FDR corrected^59^). In other words, individuals for whom we could more accurately decode social inferences in the fMRI why/how task were not necessarily participants with more social contacts.

Additional post-hoc tests also examined whether conventional univariate measures of brain responses in the why/how task—that focus on estimated neural activity in single voxels rather than multivoxel activation patterns—reflect individual differences in the number of social contacts (SNI scores). To this end, for every participant, we estimated the average univariate activation during social inferences for each ROI (based on the contrast of [why—how] inferences from participant-specific GLMs, see Methods). Correlations with SNI scores did not yield significant results in the right pSTS or any other ROI (all *p*’s > 0.31, FDR corrected^59^). These findings suggest that information predictive of social function (like SNI scores) can be obtained from distributed neural activations patterns, but not from proportionally stronger—or weaker—overall univariate activation in these localized brain areas during social inferences.

### Domain-specificity of neural predictions of the number of social contacts

Next, we tested whether the ability to predict the number of social contacts is specific to social inference activations in the brain or extends to neural markers obtained during nonsocial inferences. In other words, if participants engage in inferences that are not primarily social in nature, can we still predict variance in individuals’ SNI scores from their brain data? To address this question, we conducted a supplemental analysis of data from a nonsocial inference condition in the why/how task we had collected for the RS1 and RS2 groups (the DS group did not perform this control condition, Table 5). In this condition of the why/how task, participants made why/how inferences regarding nonsocial stimuli (e.g., inferring that a rainstorm causes water to pour out of a gutter vs. describing water pouring out of a gutter)^48^. Importantly, these supplemental analyses did not yield significant predictions of SNI scores for the right pSTS (RS1: *r* = −0.45 [−0.57, 0.47], *R*^2^ = 0.20; *p* = 0.88; RS2: *r* = −0.17 [−0.38, 0.28], *R*^2^ = 0.03, *p* = 0.72). For completeness, we repeated the analysis for the other ROIs, none of which was significant (all p’s > 0.23 in RS1 and RS2, FDR corrected for each sample). Overall, these findings suggest that neural information predictive of variability in metrics of social network size (SNI scores) is specifically related to making social inferences rather than general causal inferences.

**Table 5:** Details of how the why/how task, fMRI data collection, and image preprocessing varied across participant samples

	Discovery sample (DS)	Replication sample 1 (RS1)	Autism spectrum sample (ASD)	Replication sample 2 (RS2)
Why/how task				
Nonsocial condition	No	Yes	Yes	Yes
Blocks per condition	4	6	6	6
Stimuli per block	8	8	8	9
Total runtime (min)	5:12	16:35	16:35	13:25
Source code	https://github.com/spunt/whyhowlocalizer^104^	https://osf.io/59cbe/	https://osf.io/59cbe/	https://github.com/adolphslab/LOI2^105^
fMRI data acquisition				
MR scanner	TRIO	TRIO	TRIO	PRISMA
MR EPI sequence	Multi-band	Single-band	Single-band	Multi-band
fMRI data preprocessing				
Source code	House code^106^	House code^106^	House code^106^	fMRIPrep 1.5.3 https://fmriprep.org/en/stable/
ICA (independent component analysis)	No	No	No	Yes

Following up on evidence of domain-specificity, we also aimed to predict behavioral indices that were not explicitly linked to the social domain. More precisely, we repeated our neural decoding analysis with one major difference: we predicted variance in participants’ intelligence scores instead of the number of social contacts (Full-Scale IQ assessed in the WASI-II; see Methods) (Table 1). Neural activation patterns in the pSTS obtained during social inferences did not predict variance in IQ scores in DS, RS1, or RS2 (all *p*’s > 0.62). Complementary tests for any of the other ROIs also yielded no significant predictions of IQ scores (all *p*’s > 0.50, FDR corrected). This finding adds further specificity and, together with our inability to predict SNI scores from nonsocial inference activations in the brain, argues for discriminative validity: neural responses designed to capture a social cognitive process (social inferences) only predict aspects of social behavior.

### Predictions of individual differences in AQ and social responsiveness scores

In this set of analyses, we examined whether the pattern of activation during the why/how task might be predictive also of other behavioral indices of interest in the social domain: autism-like traits in the general population (AQ; Social Responsiveness Scales [SRS-2]). Specifically, in two separate tests, we examined whether neural activation patterns in the right pSTS obtained in the why/how task might also predict individual differences in AQ questionnaire scores^51^ in DS and RS1 and variance in SRS-2 scores^52^ in RS2. SRS-2 scores were chosen for our second replication sample for two reasons: first, this measure has been suggested to be preferable to the AQ for assessing the Broad Autism Phenotype^53^. Second, significant predictions for variance in yet another measure of autism-like traits in the general population provide further evidence that neural predictive information generalizes beyond measures of the number of social contacts (or AQ scores). The implemented cross-validated analysis was identical to the one reported above for SNI scores, simply replacing these with AQ scores (collapsed across DS and RS1) or SRS-2 scores in RS2 (Fig. 2b). Neural activation patterns of social inference in the pSTS reliably predicted AQ scores in DS and RS1 (*r* = 0.29 [−0.34, 0.24], *R*^2^ = 0.09, *p* = 0.021, permutation test, Fig. 2d) and SRS-2 scores in RS2 (*r* = 0.29 [-0.41, 0.28], *R*^2^ = 0.09, *p* = 0.045, permutation test). Notably, AQ and SNI scores were not correlated in DS and RS1 (*r* = 0.16 [−0.06, 0.37], *p* = 0.15). This suggests that predictions of variance in autism-like traits in neurotypical individuals are unlikely to merely reflect covariation of the behavioral scores of interest. Overall, the successful prediction of variance in autism-like traits indicates that predictive neural information in the right pSTS is not specifically related to SNI scores but extends to other measures of human sociability in neurotypical individuals.

### Neural processing of social inferences in the why/how task in ASD

This set of analyses aimed to extend the neural prediction of variance in social network characteristics in a sample of high-functioning adults diagnosed with ASD. First, as a sanity check, we confirmed that the neural activation patterns obtained in the why/how task decoded social inferences in the ASD group for each ROI in Table 2 (all *p*’s <0.05, FDR-corrected, ROI-wise permutation tests, Table 4, Fig. 1c). This result demonstrates that our ROIs—identified in the neurotypical DS group—also contained information for decoding social inferences in the ASD sample.

Second, we also explored whether the ROIs are equally informative about social inferences in the ASD group as they are in neurotypical individuals. For each ROI, we compared the decoding accuracies of social inferences in the why/how task between the ASD and the matched RS1 groups (see Methods). Decoding accuracies for social inferences did not significantly differ across the ASD and RS1 groups in any ROI (two-sample *t* tests, all FDR corrected *p*’s > 0.12). We also compared whole-brain accuracy maps of a searchlight decoding of social inferences in the why/how task (ASD vs. RS1, two-sample *t* test at the group level as implemented in SPM12). No brain area encoded social inferences differently for the ASD group compared to the RS1 group (even at slightly more liberal statistical thresholds of *p* < 0.001 at the voxel level, *p* < 0.05 FWE corrected at the cluster level, as implemented in SPM12). Together, these results suggest that local neural information content about social inference processing in the why/how task is not systematically diminished in ASD compared to matched neurotypical individuals in RS1.

Third, supplemental analyses also tested whether ASD and RS1 groups use similar neural activation patterns to encode social inferences in the why/how task. This was an important sanity check for our cross-sample prediction of indices of social functioning (see below). To explicitly test for shared neural representations in the why/how task across both groups, we trained the classifier on the why/how data of one group (ASD) and tested whether we could correctly identify social inferences in the other group (RS1), and vice versa (two-fold cross-validation, cross-sample decoding). For each ROI, we found that response patterns obtained in one group predicted social inference processing in the other group (*p* < 0.05, permutation tests, FDR corrected) (Supplemental Table S7). This finding suggests that—at least part of—the neural representation of social inference generalizes between the ASD and the RS1 groups.

Taken together, the results of these sanity checks suggest that ASD and a matched group of neurotypical individuals (RS1) recruit similar brain regions during social inferences in the why/how task, with matching levels of neural information content (as captured by decoding accuracies), and—at least in part—shared activation patterns. This pre-requisite, in turn, made possible the subsequent investigation outlined below.

### Prediction of number of social contacts and autism symptom severity in ASD (SNI and ADOS SA scores)

So far, we have shown that neural activation patterns of social inference processing in the pSTS predict variance in SNI scores and autism-like traits in neurotypical individuals. We next explored whether this predictive information generalized to individuals in the autism group. To this end, we used a cross-sample prediction approach: we trained our model on data from all three neurotypical participant samples (using neural activation patterns in the pSTS as features and SNI scores of 126 individuals as labels) and tested the model on data from the ASD group (using pSTS response patterns and SNI scores of individuals with autism). This approach is the most direct test of generalization of predictive neural information between neurotypical and autism groups. It also addresses potential methodological concerns related to the smaller sample size in the ASD group (i.e., concerns of limited training data in a leave-one-participant-out cross-validation approach in a small participant sample). The cross-sample prediction analysis allowed us to predict the number of social contacts in the ASD group (*r* = 0.38 [−0.35, 0.35], *R*^2^ = 0.14, *p* = 0.037, permutation test, Fig. 2e), suggesting that—at least part of—the predictive information encoded in the pSTS during social inferences generalizes from neurotypical participant groups to the ASD group.

Next, we tested whether the neural information in pSTS generalizes to other behavioral indices of social functioning in the ASD sample, namely symptom severity scores in the social domain (ADOS SA scores). To this end, we repeated the cross-sample prediction analysis with one difference: We replaced the SNI scores in the training data (neurotypical) with participants’ autism-like trait scores and the test data (ASD) with autism symptom severity scores. We found that pSTS responses in neurotypical individuals predicted variance in autism symptom severity scores in the ASD sample (*r* = 0.60 [−0.36, 0.36], *R*^2^ = 0.36, *p* = 0.001, Fig. 2f). Note that ADOS SA scores and SNI scores in the ASD group were not significantly correlated (*r* = 0.04 [−0.40, 0.46], *p* = 0.86), indicating that positive predictions of autism symptom severity in the social domain are not merely due to shared variance between ADOS and SNI scores.

Supplementary post hoc tests examined the specificity of these predictions for the social domain. We found that pSTS activation patterns did not predict variance in repetitive behaviors in the ASD group (ADOS RRB) (*r* = −0.23 [−0.35, 0.38], *R*^2^ = 0.05, *p* = 0.83). Thus, predictive information in the pSTS was specific for variance in symptom severity in the social affective domain (ADOS SA). Next, we used pSTS activation patterns obtained during nonsocial inferences in the why/how task. Neither the prediction for SNI scores (*r* = 0.30 [−0.34, 0.35], *p* = 0.92) nor ADOS-SA scores (*r* = −0.09 [−0.37, 0.37], *p* = 0.63) yielded significant results here. These results suggest that predictions of SNI scores and autism symptom severity scores in the social affective domain in the ASD group might be specific to neural activation patterns in the social domain (social inferences).

## Discussion

We found that patterns of brain responses to social inferences measured in the laboratory predict individual differences in the number of real-life social contacts measured with the SNI. This finding held across four independent participant samples (DS, RS1, RS2, ASD) when using a fixed set of functionally defined brain regions of interest (ROIs), pointing to the robustness and generalizability of our key finding. The number of social relationships measured with the SNI is a metric that has received considerable attention in the literature, in both behavioral and neuroimaging studies^10–12,14,15,20,22–26,36,60^. Our results directly link peoples’ capacity to engage in a specific social cognitive process (thinking about other people’s inner mental states) and individual differences in real-world social behavior (yielding varying social network sizes). We found remarkable specificity for a particular neuroanatomical region (the right pSTS). At the same time, our findings generalized across multiple measures of individual differences in social behavior (SNI scores, autism-like traits in neurotypical individuals in community samples (AQ, SRS-2), and autism severity scores (ADOS SA)). We suggest that the patterns of neural activations in the pSTS during social inference reflect an underlying latent variable that comes into play relatively ubiquitously in real-life social behavior. Our findings further argue that variability in the number of social relationships has a partly neural basis. Moreover, the results point to the potential of using process-specific neural markers to characterize individual differences in real-world social behavior.

We predicted the number of social contacts from brain activations during a laboratory task that required inferring other people’s mental states (e.g., their feelings, thoughts, or intentions). Such social inferential processing is often referred to as “mentalizing” or “theory of mind”^29,61,62^, encompassing a rather broad collection of different abilities^44,63^ and recruiting a network of multiple brain regions^41–43^. Our present findings provide considerable further specificity on how these processes are associated with social behavior. Notably, we found high neuroanatomical specificity: while multiple brain regions decoded social inferences in the why/how task (Table 2), information that was predictive of variance in the number of social contacts (as well autism-like traits) was exclusively localized to the right pSTS (Table 3). The anatomical specificity of our predictions suggests that different nodes of the brain network recruited during social inferences might implement distinct mental computations, and only the computations in the pSTS generalize to indices of social functioning tested in our study. Our analyses shed light on the nature of the predictive neural computations in the pSTS: brain responses obtained during why/how inferences for nonsocial stimuli (e.g., related to extreme weather phenomena) did not predict the number of social contacts, autism-like traits in neurotypical individuals, or symptom severity in our autism group. This finding suggests that information predictive of the number of social contacts is not coming from domain-general computations of making high-level inferences, but specifically from inferences about other people’s mental states. These findings fit emerging evidence for the range of social processes subserved by the STS. In particular, there is an anterior-to-posterior organization that localizes processes ranging, respectively, from language to voices to faces to biological motion to theory-of-mind^64,65^. The region we found in our study, the very posterior STS, corresponds to the one most specifically implemented in abstract social inferences^65^ (Figure S2a). Finally, neural activation patterns for social inferences in the why/how task did not predict variance in people’s cognitive functioning that was not necessarily social in nature, such as intelligence scores or symptom severity regarding restrictive and repetitive behaviors in the ASD group (ADOS RRB scores). This finding further supports the notion of the high specificity of our neural prediction to differences in people’s social behavior: neural responses capturing a social cognitive process (social inferences) only predict aspects of social behavior, arguing for discriminant validity. Overall, our results demonstrate the potential of using process-specific, localized neurobiomarkers of cognitive abilities that contribute to social behavior as robust predictors of people’s social functioning in the real world.

At the same time, we demonstrated that predictive neural information generalizes to other indices of people’s social functioning. Specifically, we could also predict variance in autism-like traits in the general population (AQ and SRS-2 scores) and autistic social symptom severity in the ASD group (ADOS SA scores). This generalizability is especially important given that our primary measure of social network size, the SNI score, is based on self-reports. The ADOS SA scores, by contrast, are based on observer ratings of behavior, arguing that our findings are not merely driven by response biases on questionnaires. The convergence of associations we found, and the fact that they pertain specifically to the activation patterns produced by social inference processes, go well beyond prior findings based on resting-state or structural MRI data. They also raise the important question of what exactly drives our findings. Although we have previously argued for caution in studies that claim to find associations between the SNI and brain structure^13^, variance in brain structure in the right pSTS (namely gray matter density) has previously been linked to variance in the number of friends on online social network services^12^. Predictions of indices of social behavior unrelated to metrics of social network size point to the intriguing possibility that social inference activation in the pSTS reflects a latent variable that predicts individual differences in social functioning quite broadly (such as AQ and SRS-2 scores, ADOS SA severity scores, and the number of social contacts). It remains an open question how far this generalizability would extend, e.g., to more sophisticated metrics of social networks such as social network position, characteristics of social ties or social distance^22,66,67^, or even other social behaviors known to activate similar brain regions of the pSTS such as altruistic behavior^35,56^ (see Figure S2b for illustration of overlap). Multi-trait-multi-method approaches (MTMM)^68^ might be well suited to address these future questions, shedding further light on the underlying latent variable that we propose and on how the cognitive processes engaged could be causally related to social connectedness and everyday social functioning.

Another key question about generalizability is whether our findings would extend to different participant samples, specifically ones not necessarily representative of the discovery sample, either in terms of predictor variables (i.e., the cognitive process of interest or its neural signature in the brain), the dependent variable (i.e., markers of real-world social functioning and connectedness), or both. We explicitly tested the generalizability of our findings in a sample of high-functioning individuals with autism. Marked deficits in social interaction and specifically in understanding other people’s mental states are a hallmark of ASD^39^. Yet, we found that brain responses of social inference measured in the lab predict the number of social contacts, and metrics of autism symptomatology in the social domain (ADOS SA). Notably, the successful predictive model was trained on data from neurotypical individuals. This approach is the most direct test of generalization of predictive neural information between neurotypical and ASD groups. Our results point toward a generic component of information encoded in activation patterns pSTS that is shared across neurotypical and the ASD sample. Overall, the findings suggest that neural predictions of social behavior extend to individuals in non-representative samples with atypical behavior in the social domain.

Evidence of successful cross-sample predictions of social functioning in ASD has important practical implications: limited sample sizes in special interest groups or clinical populations are common, highlighting the broad appeal of using neurotypical groups—that are often easier to recruit and test—to train predictive models of interest. Moreover, on a conceptual level, our results support the idea that social inference abilities are continuously distributed across neurotypical individuals drawn from a community sample and neurodiverse individuals with ASD. Instead of a categorically defined condition, ASD can be viewed as one end of a continuous distribution of core competencies and/or deficits that occur in nature^69^ (but see ref. ^70^). Our findings across neurotypical and ASD groups support an understanding of the core feature of problems with social interactions of ASD as a quantitative trait rather than a categorically defined condition^69^, with implications for diagnostic classification. Our findings also suggest that neural activations to social inference could be incorporated into developing biomarkers for ASD, offering avenues for diagnosis and mechanistically targeted treatments^71^. Biomarkers for autism are scarce, partly due to the heterogeneity in phenotypes and difficulties in identifying “biological tests” to predict treatment responses^72^. Neural measures of atypical cognitive processes, such as the ones we feature in this study, hold the promise of predictive biomarkers sensitive to quantitative variations in core features of ASD.

While reliable across four participant samples, some limitations and open questions need highlighting. For instance, our results remain limited by small sample sizes, reducing our power to detect possible effects in this study. Thus, the lack of prediction from ROIs other than the right pSTS may have resulted partly from small sample sizes. Future replications in much larger samples that test hundreds or thousands of individuals will be necessary to establish the likelihood of any negative findings further. Likewise, while our results show a link between social inferences and social network characteristics, they can’t speak to variance in other social behaviors (e.g., normative choices^73^ or expressions of prosocial phenotypes^74–76^).

Another important open question concerns the trajectories of neural prediction of social network characteristics over time. ASD is a neurodevelopmental disorder that arises early in life, raising the question of how the predictive information of our identified neural markers changes across the lifespan. Do these markers characterize social deficits when they are first displayed? Can they predict symptom severity later in life or identify subgroups of young children likely to respond to treatments targeting socio-cognitive processing? Likewise, in neurotypical individuals in the general population, social network characteristics change over time. Longitudinal studies will be necessary to assess the predictive value of functional neural markers across time. It will be interesting in this respect also to investigate the effects of training people’s social skills^77,78^ to see how the relationship between neural markers and social networks can be modified.

These considerations raise the perhaps most challenging question about interpreting our findings: their causal relation. Do social inference skills, as indexed in pSTS activation patterns, cause real-world social behavior that influences social network size? Or do people with larger social networks and more social contacts in daily lives develop better social inference skills? In general, it is surprising that there should be any substantial relation at all since social networks are influenced by many factors in life that appear independent of social inference skills (moving to a new location, time available to socialize, type of job, etc.). The only clear evidence for a causal relationship currently comes from studies in Macaques where social membership to groups of variable size could be experimentally assigned^79^. Living in larger social groups *caused* changes in brain structures as measured in increased gray matter volume and changes in brain function in non-human primates^79^. Positive correlations between a monkey’s social rank within a group and neuroanatomical markers support the notion that social structure is associated with neural measures. However, these results leave unclear the precise cognitive processes mediating between the two. This evidence would argue that the vagaries of life circumstances cause membership in a particular social group, and this, in turn, causes changes in the neural substrates of social cognition. More in-depth characterization of people’s life variables in a longitudinal design could shed further light on these intriguing questions.

It is implausible that the pattern of brain activation and SNI scores we observed in our study are directly causally related. After all, while we acquired neuroimaging data, participants were neither filling out SNI questionnaires nor otherwise instructed to think about their friends or social networks. So what could explain the reliable association that we found? An alternative possibility is that both brain activation patterns and the number of real-world social relationships measured by SNI scores are caused by a shared latent variable: the social cognitive processes engaged during our activation task. Considering the task conditions that elicit the raw data that constituted the predictive multivoxel activation patterns, we propose that it is precisely the intended social inference process that constitutes the latent variable in question. Participants are thinking about other people’s mental states and the intentions responsible for the actions depicted in the stimuli, and it is such mental state attribution that is causally related to establishing larger numbers of social relationships (as indexed with the SNI).

This hypothesis is supported by the relationship we found with SRS, AQ, and ADOS severity scores, particularly by the fact that AQ scores are not themselves correlated with SNI scores. Instead, these two metrics of social functioning (autism-like traits as captured in AQ and SNI) are independently associated with brain activation patterns. Our interpretation is that social inference must itself be comprised of multiple cognitive sub-processes, distinct sets of which are causally related to different consequences in the social world: autism-like traits and SNI. Although it is known that social inference is not monolithic^44,63^, this still leaves open considerable detail to be investigated. The anatomical specificity we found will be particularly informative in further dissecting this issue in future studies. For instance, our prior work has shown that social inference activation can be correlated with social curiosity or attributional complexity skill—but in this instance, the effect was found only in the dmPFC, not in the pSTS^48^. Our findings emphasize the pSTS as one node in the social cognition network whose variable activation patterns are a promising marker for social functioning in the real world.

## Method

### Participants

The study reports data from four participant samples: the discovery sample (DS, *n* = 60) and two replication samples (RS1: *n* = 20; RS2: *n* = 55) of neurotypical volunteers from the Los Angeles metropolitan and the group of high-functioning individuals diagnosed with autism spectrum disorder (ASD, *n* = 25) (see Table 1). Individuals in the RS1 group were age-, sex- and IQ-matched to individuals in the ASD group to allow for a matched comparison of neural activation of social inferences (why/how task) across groups. All participants in the ASD group had a prior clinical diagnosis of autism spectrum disorder, which was confirmed by revised algorithm scores on the Autism Diagnostic Observation Schedule (ADOS), Module 4. All participants (DS, RS1, RS2, ASD) participated in a version of the why/how fMRI task (Table 5) and a separate behavioral session in exchange for financial compensation ($20/h). All participants were right-handed, had a normal or corrected-to-normal vision, spoke English fluently, and had IQ scores in the normal range (as assessed using the Wechsler Abbreviated Scales of Intelligence-II; FSIQ, Table 1). Sex at birth was assessed using self-reports (see Table 1; source data are also reported in a de-identified form on OSF, 10.17605/OSF.IO/RNT8S). Sex differences were not hypothesized for the current research question. We excluded data from one participant in the DS group and one participant in the RS2 group due to poor performance in the why/how fMRI task (no responses to > 70% of trials). We excluded data from two individuals in the ASD group due to artifacts in the fMRI data. We excluded data from one participant in RS2 due to an SNI score of 106 that exceeded three standard deviations above the group average (Table 1) and another three individuals in the RS2 group due to excessive motion during the why/how task. Motion outliers at the individual level were identified and excluded based on the approach described in ref. ^80,81^, which uses low-pass filtering to control false rejections associated with respiration and pseudo-motion signals present in short repetition time multi-band EPI sequences. The six rigid body motion parameters estimated during pre-preprocessing were low-pass filtered temporally using a fifth-order Butterworth filter with a critical frequency of 0.2 Hz. Following filtering, the framewise displacement (FD) time series were calculated^82^ for each participant. Participants with unusually high motion were identified from the set of individual 50^th^ and 95^th^ percentile frame-wise displacement (FD) values using the DBSCAN clustering algorithm^83^ implemented by scikit-learn^84^. All participants provided written informed consent according to a protocol approved by the Institutional Review Board of the California Institute of Technology (#12−0343).

### Behavioral indices of social functioning

Social network characteristics (number of social contacts, SNI): We measured participants’ social network characteristics using the SNI^4,15,26^. The questionnaire assesses how many people participants see or talk to regularly in 12 types of social relationships, including family, friends, workmates, neighbors, religious groups, etc. (for a complete list, see http://www.midss.org/content/social-network-index-sni). Our core measure of interest, the estimated number of social contacts, refers to the total number of people with whom the respondent has regular contact at least once every two weeks, regardless of the type of social role involved. While not the focus of our study, the SNI also yields two additional scores: network diversity (number of unique social roles in which the participant regularly interacts with at least one other person) and number of embedded networks (number of different network domains in which a respondent is active).

Autism-like traits in neurotypical individuals in DS, RS1, and RS2 (AQ and SRS-2): Within the general population, autism-like traits have been proposed to be continuously distributed^52^. We measured variance in autism-like traits using the AQ^85^ (Autism Spectrum Quotient; in DS, RS1) and Social Responsiveness Scale-2^52^ (SRS-2; in RS2). Both the AQ and SRS-2 have self-report versions used to screen for ASD in adults.

The Autism Spectrum Quotient (AQ) is a self-report survey used to screen for autism spectrum traits in adults with normal-range intelligence (https://www.autismresearchcentre.com/arc_tests). It is comprised of 50 items scored using a 4-point Likert scale to indicate how strongly an individual agrees or disagrees with each statement (e.g., “I find it difficult to work out people’s intentions”; “I find myself drawn more strongly to people than to things”). Scores at or above 32 are a strong indicator that autism symptoms are present. Note, however, that AQ scores are not designed for diagnostic purposes.

The Social Responsiveness Scale-2 (SRS-2), Adult Form, Self-Report is a 65-item questionnaire that assesses social deficits associated with autism spectrum disorder. It is sensitive to mild forms of impairment in individuals who do not meet the criteria for an autism diagnosis, as well as to variance in the severity of deficits among individuals with ASD. We have collected the SRS-2 for our second neurotypical replication sample (RS2) as the SRS-2 has been suggested to be preferable to the AQ for assessing the Broad Autism Phenotype^53^. It also allowed assessing if our results could be replicated using yet another behavioral measure of variance in atypical social processing associated with autism.

Symptom severity scores in ASD (ADOS SA): ADOS SA scores served as a quantitative, continuous measure of autistic symptom severity in the social affective domain in the ASD group. The Autism Diagnostic Observation Schedule, 2nd Edition (ADOS-2^86^), is a standardized observational diagnostic measure. ADOS-2 Module 4, designed for verbally fluent adolescents and adults, was administered to all participants with a prior diagnosis of ASD or clinically significant social skills deficits (i.e., social skills deficits that required clinical treatment in the PEERS program at UCLA; https://www.semel.ucla.edu/peers/young-adults). Following the ADOS-2 protocol, a clinically trained examiner engaged each participant in a series of standardized discussions and tasks and rated specific aspects of participant’s communication and social behavior on a scale from 0 (no abnormality) to 3 (severe abnormality). The revised diagnostic algorithm^55^ was used to score the presence of autistic behaviors in the Social Affect (SA) domain and Restrictive and Repetitive Behaviors (RRB). The ADOS SA score provides a classification of autism spectrum disorder or nonspectrum, with a sensitivity of 89% and specificity of 72.2%. The algorithm scores were then converted to a calibrated measure of severity, which provides a continuous, quantitative index of social-communication (SA) and restricted and repetitive behaviors (RRB) extending beyond the diagnostic categorization of autism^54^. Given our focus on indices of social functioning, the analyses focused on ADOS SA scores.

### Functional brain markers of social inference: why/how task (fMRI)

To assess functional neural markers of social inference processing, participants in the DS performed an established why/how social inference task^47,48^ during fMRI data acquisition (see Table 5 for the hyperlink to source code). The task uses a 2 (inference: why, how) × 2 (target stimuli: faces, hands) factorial design implemented in pseudo-randomized blocks (4 blocks per condition). Each task block consists of a series of 8 images that display social targets in the form of emotional facial expressions (faces) or intentional hand actions (hands) (Fig. 1a). Each image requires a speeded judgment (yes/no), entailing either social inferences about others’ internal states (“why?”; high level of inference, e.g., “Is the person helping someone?”) or factual inferences about the observed behavior (“how?”; low level of inference, e.g., “Is the person looking to their side?”). Table S3 presents a complete list of block-specific questions. Each image is presented twice, once per inference condition. A brief verbal cue (e.g., “helping?”) presented prior to every target image after the first minimizes working memory demands. Participants had a maximum of 1750 ms to respond before the presentation of the next image. Once an answer was given, the task immediately advanced. Block onsets were fixed across participants. The order and onsets of trials were optimized to maximize the efficiency of separately estimating the contrast of interest [why > how] for each of the two target categories. In addition, the order of why and how blocks were counterbalanced within each target category. The order of trials was achieved by using open-source fMRI design optimization software (https://zenodo.org/record/58616#.W5muJWaUnbk) to generate one million pseudo-random designs and for each summing the efficiencies of the contrasts of interest. The most efficient design for each task was retained and used for all participants. Stimulus presentation and response recording used the Psychophysics Toolbox PTB 3.0.13–3.0.16^87^. An LCD projector was used to present the task on a screen at the rear of the scanner bore that was visible to participants through a mirror positioned on the head coil. Participants were given a button box and made their responses using their right-hand index and middle fingers.

Performance in the why/how task was assessed as follows: for each participant, we computed measures of mean percent accuracy, d’ and response time (RT) for the four conditions (inference [why, how] × targets [faces, hands]). We omitted rare trials with no response (mean = 0.46%, max = 3.91%, across participants). To address negative skewness, accuracy scores were subjected to a Box–Cox transformation before undergoing statistical testing^88^. Mean RTs were computed after excluding values greater than three SDs from the mean. Table S2 reports group-level descriptive statistics.

The version of the why/how social inference task performed by participants in the RS1, RS2, and ASD groups differed in several ways from the one introduced above. These differences were motivated by the specific questions being investigated in the larger study for which it was designed. First, and most importantly, in addition to social target stimuli (hands, faces; Fig. 1a), modified task versions featured a third stimulus category showing the effects of nonsocial processes (e.g., scenes showing the consequences of extreme weather) (for details, see ref. ^48^). Matching the social stimulus conditions (faces, hands), each nonsocial stimulus was presented twice, requiring either a high level of inference (“why?”) or a low-level inference based on facts displayed in the picture (“how?”). Note that data from this nonsocial control condition was outside of the focus of the current study and was only analyzed for post hoc analyses that examined the specificity of the neural prediction of social functioning (e.g., number of social contacts) for making inferences about other people’s inner mental states. Second, task versions differed regarding the number of trials per block and the number of blocks (Table 5). Third, block-specific questions differed slightly (Table S3). Fourth, there were minor differences in the timing of the trial elements within each block and with the average stimulus onset asynchrony^48^ (see Table 5 for further details and hyperlink to source code).

### Functional image acquisition (fMRI)

All imaging data were acquired at the Caltech Brain Imaging Center. Imaging data of DS, RS1, and ASD were collected using a Siemens Trio 3.0 Tesla MRI scanner outfitted with a 32-channel phased-array head coil. For the DS, we acquired 304 whole-brain T2*-weighted echoplanar image volumes (EPIs; voxel resolution = 2.5 × 2.5 × 2.5 mm^3^, 56 slices, TR = 1000 ms, TE = 30 ms, flip angle = 60°, FOV = 200 mm, interleaved acquisition order, multi-band acceleration factor = 4) for the why/how social inference task. For the RS1 and ASD groups, whole-brain T2*-weighted EPI volumes for the why/how task were acquired with the following MR protocol: voxel resolution = 3 × 3 × 3 mm^3^, 47 slices, TR = 2500 ms, TE = 30 ms, flip angle = 85°, FOV = 192 mm, ascending acquisition order. For RS2, imaging data were collected at Siemens 3.0 Tesla MAGNETOM Prisma.Fit MRI scanner outfitted with a 32-channel phased-array head coil. For RS2, we acquired 1080 whole-brain T2*-weighted EPI volumes with the following parameters: TR = 700 ms, TE = 30 ms, 60 contiguous oblique transverse slices, slice pitch 20 degrees, 2.5 mm isotropic voxel size, multi-band acceleration = 6, interleaved slice acquisition order, flip angle = 53 degrees. A pair of spin echo EPI volumes (TR = 5500 ms, TE = 48 ms, and multi-band acceleration = 1) with opposing phase encoding polarity were acquired for geometric distortion correction with identical geometry and EPI echo spacing to the T2*-weighted EPI volumes. Participants’ in-scan head motion was minimal (max translation = 2.78 mm, max rotation = 1.88°), and movement parameters were comparable for RS1 and ASDs (no significant between-group difference in frame-to-frame displacement or frame-to-frame rotations as measures of participant motion most likely to cause artifactual BOLD signal correlations; unpaired *t* tests, *p*’s > 0.17, uncorrected). For all participants, we also acquired a high-resolution anatomical T1-weighted image (1 mm isotropic) and field maps used to estimate and correct for inhomogeneity-induced image distortion.

### Image preprocessing (fMRI data analysis)

Images in three participant samples (DS, RS1, ASD) were processed primarily using Statistical Parametric Mapping (SPM12, http://www.fil.ion.ucl.ac.uk/spm) together with house code (see Table 5 for the hyperlinks). Prior to statistical analysis, functional imaging data were subjected to the following preprocessing steps: (1) the first four EPI volumes were discarded to account for T1-equilibration effects; (2) slice-timing correction was applied; (3) the realign and unwarp procedure was used to perform distortion correction and concurrent motion correction; (4) the participants’ T1 structural volume was co-registered to the mean of the corrected EPI volumes; (5) the group-wise DARTEL registration method included in SPM12^89^ was used to normalize the T1 structural volume to a common group-specific space, with subsequent affine registration to Montreal Neurological Institute (MNI) space; (6) all EPI volumes were normalized to MNI space using the deformation flow fields generated in the previous step, which simultaneously re-sampled volumes to 2 mm isotropic, (7) and smoothed using a Gaussian kernel of 6 mm isotropic, full width at half maximum (FWHM).

Images in RS2 were obtained after a major update of the MR scanner and were preprocessed using the open-source fMRIPrep 1.5.3 analysis pipeline^90^, which utilizes a combination of tools from well-known software packages (see Table 5 for hyperlink). FMRIPrep is designed to provide an easily accessible, standardized, state-of-the-art fMRI data preprocessing pipeline that is robust (e.g., to variations in-scan acquisition protocols) and requires minimal user input. An extensive description of the analysis steps is provided in the boilerplate that fMRIPrep generates for every participant processed (Methods S1).

### GLM: block-wise estimates of social (‘why?’) and factual (‘how?’) inferences in the why/how fMRI task

For each participant, a general linear model (GLM) estimated regressors of interest for each block of the why/how task. Blocks were defined by the onset of the first target image and the offset of the final image of the block^91^. The GLM included as covariates of no interest the six motion parameters estimated from image realignment and a predictor for every time point where the in-brain frame-wise signal change (calculated as the root mean square derivate, or DVARS) exceeded 2.5 SDs of the mean DVARS across the time series or where frame-wise displacement exceeded 0.5 mm of translation or 0.5° of rotation^92^. Note that participant-wise GLMs in RS2 only included as covariates of no interest the six motion parameters estimated from image realignment due to differences in the preprocessing pipeline in this sample (Table 5, Methods S1). The hemodynamic response was modeled using the canonical (double-gamma) response function and a 1/100 Hz high-pass cutoff filter to eliminate low-frequency drifts in data. GLMs were estimated using the SPM12 RobustWLS toolbox, which implements the robust weighted least-squares estimation algorithm^93^. Estimated responses for the block-wise regressors of interest (i.e., ‘why’ and ‘how’ inference task blocks) were used as inputs for the multivariate classification of ‘why’ vs. ‘how’ inferences to identify (DS) or confirm (RS1, RS2, ASD) brain regions that decode social inferences in the why/how task (described below). Moreover, for each participant, we estimated one contrast image based on regressors of interest in conditions that included social stimuli ([why _face-blocks_ AND why _hand-blocks_]—[how _face-blocks_ AND how _hand-blocks_]). These participant-specific contrast images were used as inputs for the multivariate regression analyses to predict variance in participants’ number of social contacts (SNI scores) (and other behavioral indices of interest such as AQ, SRS-2, or ADOS SA scores). For each participant, we also estimated one matching contrast image for the nonsocial control condition [why _nonsocial_—how _nonsocial_]. Note that this contrast was only estimated for participant samples for which the nonsocial task condition was available (Table 5). These participant-wise contrast images were used as input for the post-hoc multivariate regression analysis that probed the social specificity of our neural predictions.

### Identifying ROIs of social inference processing in the why/how task in DS

In the first step, we identified brain regions that are reliably engaged during social inference processing in neurotypical individuals in the DS group. More precisely, this initial analysis aimed to identify multi-voxel activation patterns in the brain that decode social (versus factual) inferences in the why/how task (i.e., why versus how task blocks). To this end, we used a linear support vector machine classifier (libSVM, http://www.csie.ntu.edu.tw/~cjlin/libsvm) in combination with a standard whole-brain searchlight approach^49,94,95^ implemented in MATLAB 2021a). This approach has been used in various domains of cognitive and social neuroscience^95–97^. This data-driven approach does not depend on a priori assumptions about informative brain regions and ensures unbiased information mapping throughout the whole brain.

For each participant, we defined a sphere with a radius of four voxels around a given voxel v_i_ of the measured brain volume^56,98–100^. We extracted the parameter estimates of the *N* voxels within this sphere from the block-wise regressors of interest from the GLM (see above). The resulting N-dimensional pattern vectors were created separately for each block and condition of the why/how task (inferences [why, how] × target [hands, faces]). We then trained a linear kernel support vector machine classifier to distinguish neural pattern vectors associated with either social or factual inferences (why versus how; fixed cost parameter *c* = 1). Training data consisted of all but one neural pattern vector of a particular inference condition obtained for the participant (i.e., leave-two-blocks-out cross-validation; left-out-data: neural pattern vectors for one why block and one how block). The resulting model provided the basis for the prediction of the inference condition of the two left-out task blocks (test data) solely based on their neural pattern vectors. This procedure was repeated several times, always using pattern vectors of a different task block as test data. Predictive information was defined as the average percentage of correct classification of the inference condition and was assigned to the central voxel of the searchlight sphere. This approach was repeated for every voxel of the measured brain volume, yielding a three-dimensional decoding accuracy map for every participant. Participant-specific accuracy maps were then used in a random effect group analysis for DS (single t-test as implemented in SPM12) and tested against chance level (50% in this binary classification) (*p* < 0.05, FWE corrected at the voxel level for the whole brain volume as implemented in SPM12). To identify response patterns that decoded engagement in social inferences independent of the social stimulus triggering this process, we implemented the decoding analysis separately for each target condition of the why/how task (i.e., faces and hands, Fig. 1a, Table S4 and Table S5). This also allowed us to verify that faces and intentional hand actions elicited common inference processes in the brain^101^. At the group level, we then identified brain regions that decoded social inference across both target conditions, using the implicit masking function in SPM12 (*p* < 0.05, FWE corrected at the voxel level for the whole brain, cluster threshold of five voxels). The resulting clusters were defined as regions of interest (ROIs) for the multivariate prediction of individual differences in social network characteristics (see below) for all four participant samples. Focusing on a fixed set of brain areas identified in DS minimizes the risk of circular analysis (i.e., double dipping)^57^ and producing false positive results in the remaining three participant samples (RS1, RS2, ASD).

### Confirming ROIs of social inference processing in the why/how task in RS1, RS2, and ASD

For each ROI, we tested if neural activation patterns decoded social inferences in the why/how task in RS1, RS2, and ASD, respectively. The sanity check used the following analysis steps: for every ROI, we separately carried out a decoding analysis of social inference (why versus how) for data of the why/how task (separately for individuals in RS1, RS2, and ASD). This analysis conceptually matched the searchlight decoding analysis implemented in DS with one major difference: neural activation patterns were extracted from a particular ROI identified in DS (not a spherical searchlight cluster). Non-parametric permutation tests assessed the statistical significance of average ROI-wise decoding accuracies for each participant sample. Actual decoding accuracies for the group were compared to accuracies of an empirical null distribution (realized by randomly permuting the pairing of participants’ neural pattern vectors and the binary label coding for the inference condition, fixed permutation order across participants, 1000 permutations). Only predictions above the 95th percentile of null distributions were considered statistically significant^102^. For each participant sample, we used the fdr_hr function in MATLAB R2021a to apply FDR correction^59^ across the family of ROI-wise statistical tests. Permutation tests were not used in the whole brain searchlight analysis in DS (see above) due to the computational costs associated with the high number of searchlights (~250,000) compared to the seven ROIs.

### Neural prediction of individual differences in the number of social contacts

Having identified multi-voxel activation patterns that reliably decoded social inference processing, we proceeded to the key question of the study: can we use functional neural markers of social inferences to predict individual differences in social network characteristics? To address this question, we ran a support vector regression (SVR) analysis of an individual’s SNI scores (labels) and the participant’s ROI-based brain responses in the why/how task (features).

First, we describe the cross-participant prediction in neurotypical groups. For each ROI (Table 2), we performed the following analysis steps: for every participant, we extracted parameter estimates for all voxels in an ROI (Fig. 1b) from the contrast image of individuals’ GLM ([why _face blocks_ AND why _hand blocks_]—[how _face-blocks_ AND how _hand-blocks_]). The resulting neural pattern vectors (one per participant) were used as input features for the prediction, and the participant’s number of social contacts (SNI scores) served as labels (z-scored for all individuals within a participant sample). To implement the multi-voxel SVR, we used a linear ν-SVR in LIBSVM (a popular library for support vector machines that has gained wide popularity in machine learning and many other areas) in MATLAB R2021a. We used a fixed cost parameter *c* = 1 and a leave-one-participant-out approach. For example, for the discovery sample DS, this approach yielded a 59-fold cross-validation: training was based on data from 58 participants (training data). We then tested if the model could predict the SNI score of the remaining participant solely based on this participant’s ROI-specific neural activation pattern (test data). ROI-specific prediction accuracies reflect correlations of the observed and predicted social network score across participants of each participant sample^35,56,99^. Permutation tests assessed the statistical significance of the prediction by comparisons to the empirical null distribution estimated for this ROI and sample (realized by randomly permuting the pairing of participants’ neural pattern vectors and behavioral SNI scores 1000 times). Only predictions above the 95th percentile of null distributions^102^ that survived FDR correction across the seven ROIs^59^ were considered statistically significant for participants in DS. FDR correction was implemented using the fdr_hr function in MATLAB R2021a. These analysis steps were repeated for data from the RS1 and RS2 groups, as well as for other indices of social functioning such as AQ, SRS-2, and WASI-II IQ scores. ROIs were identical across all four participant samples to explicitly test the generalizability of our findings in DS to data from other participant samples.

Next, we tested whether predictive neural information generalizes from neurotypical participant samples to individuals in the ASD group. To this end, we used a cross-sample approach to predict SNI scores (number of social contacts) and symptom severity in the ASD group. The analysis approach matched the cross-participant prediction described above with one significant difference: we trained our model on data from all three neurotypical participant samples (using participant-wise activation pattern vectors from the right pSTS of 126 individuals as features and their SNI scores as labels) and tested the model on data of the ASD group (using pSTS-wise activation pattern vectors of 23 individuals as features and their SNI scores as labels). Both training and test data were standardized (separately for each sample) using the z-score function in MATLAB R2021a to account for differences in the feature space across samples (e.g., due to differences in the scanner, acquisition protocol, and preprocessing of fMRI data, Table 5). Likewise, behavioral scores were standardized (*z*-scored) to account for differences in behavioral measures across samples (e.g., scales of autism-like measures in neurotypical samples and ADOS scores in the ASD group, see below). This cross-sample prediction approach is the most direct test of generalization of predictive neural information between neurotypical and autism groups. It also addresses potential methodological concerns related to the smaller sample size in the ASD group (i.e., concerns of limited training data in an alternative leave-one-participant-out cross-validation analysis approach in the ASD group). Permutation tests assessed the statistical significance of predictions. These analysis steps were repeated for the neural prediction of autism symptom severity in ASD (ADOS SA scores).

### Statistics and reproducibility

The DS, replication sample 1 (RS1), and autism sample (ASD) were based on existing data (DS = 60, RS1 = 20, ASD = 25, see Table 1), taking advantage of their study design (why/how task: block design, see Table 5) and sample sizes. We collected a separate replication sample (RS2, *n* = 55, see Table 1) of approximately the same size as DS to replicate the effects found in the DS group. No statistical method was used to predetermine sample size. Details on data exclusion criteria are provided in the Participants section. All participant samples completed a version of the why/how fMRI task and a battery of behavior measures to characterize social functioning. No randomization was used. No blinding was applied. The statistical analyses of the data are described in the context of their respective analysis and research question in the Methods (see above). All statistical tests are 2-tailed unless stated otherwise. The study contained multiple replications of the original findings in the DS, including two neurotypical replication samples (RS1, RS2) and one autism sample (ASD). For the prediction in the smaller participant sample of the ASD group, we used a cross-sample prediction approach: we trained our model on data from all neurotypical subject samples and tested the model on data from the ASD group. This approach addresses potential methodological concerns related to the smaller sample size in the ASD group.

### Reporting summary

Further information on research design is available in the Nature Portfolio Reporting Summary linked to this article.

## Supplementary information

Supplementary Information

Reporting Summary

## Data Availability

The brain data (fMRI) for all neurotypical data generated in this study are available here: Conte Social Inference and Context collection at https://nda.nih.gov/edit_collection.html?id=2643; fMRI data for the autism sample as can be found on the Open Science Framework (OSF, 10.17605/OSF.IO/RNT8S). Source data are provided in a de-identified form with this paper and on OSF (10.17605/OSF.IO/RNT8S) Source data are provided with this paper.

## Source data

Source Data

## Code availability

Behavioral and neural data were analyzed using MATLAB R2021a. FMRI data were analyzed using SPM12 (https://www.fil.ion.ucl.ac.uk/spm/); see hyperlinks in the manuscript for code regarding preprocessing and first-level GLMs; code underlying the MVPA analysis is openly available here^103^ or upon request.
