# Supplementary material for: Neural signatures of social inferences predict the number of real-life social contacts and autism severity
Source: Nat Commun. 2023 Jul 20;14:4399. doi: 10.1038/s41467-023-40078-3 (PMC10359299; doi:10.1038/s41467-023-40078-3)
Supplement: Supplementary file 1 — Supplementary Information [file 41467_2023_40078_MOESM1_ESM.pdf]

Supplemental Material

Neural signatures of social inferences predict the number of real-life social contacts and autism severity

Anita Tusche [1,2,*], Robert P. Spunt [1], Lynn K. Paul [1], Julian M. Tyszka [1], Ralph Adolphs [1,3]

[1] Division of the Humanities and Social Sciences, California Institute of Technology, Pasadena, CA 91125, U.S.A.

[2] Department of Psychology, Queen's University, Kingston, Ontario K7L 3N6, Canada

[3] Division of Biology and Biological Engineering, California Institute of Technology, Pasadena, CA 91125, U.S.A.

* Corresponding author:

Anita Tusche

Queen's University

344 Humphrey Hall

Ontario K7L 3N6, Canada

Phone: +1 (613) 533-2351

Email: anita.tusche@gmail.com

Table of Contents

Table S1. Positive inter-correlation of SNI scores in DS, RS1, RS2 and ASD 3

Table S2. Behavioral performance in the why/how task (fMRI) 4

Table S3. Block-specific question endings used in the why/how task........ 5

Table S4. Brain regions decoding social inferences in Face blocks in the why/how fMRI task in the Discovery Sample (DS) 6

Table S5. Brain regions decoding social inferences in Hand blocks in the why/how task in the Discovery Sample (DS) 7

Table S6. Univariate results of social inference [why vs how] in the Discovery Sample (DS) 8

Table S7. Shared neural code for social inferences [why vs. how] in the why/how task for ASD and RS1 (cross-sample decoding). 10

Table S8. Activation patterns in each brain region of interest (ROI) decoded social inferences 11

Figure S1. Overlap of univariate and multivariate analyses of social inferences in the why/how task in the Discovery Sample (DS). 12

Figure S2. Correspondence of our pSTS ROI with previous pSTS findings. 14

Results S1. Stimulus-specific decoding of social inferences in the why/how task in the Discovery Sample (DS). .. 15

Methods S1. Detailed description of fMRI data preprocessing in RS2 (fmriPrep) 16

Table S1. Positive inter-correlation of SNI scores in DS, RS1, RS2 and ASD

	Social Network Size	Network Diversity	# Embedded Networks
DS			
Social Network Size		0.79 ** (8.1e-14)	0.91 ** (8.8e-24)
Network Diversity			0.65 ** (2.9e-08)
# Embedded Networks			
RS1			
Social Network Size		0.68 * (0.0026)	0.83 ** (3.8e-05)
Network Diversity			0.25 (0.3341)
# Embedded Networks			
ASD			
Social Network Size		0.85 ** (2.2e-07)	0.86 ** (1.1e-07)
Network Diversity			0.57 * (0.0041)
# Embedded Networks			
RS2			
Social Network Size		0.80 ** (2.4e-12)	0.83 ** (9.1e-14)
Network Diversity			0.70 ** (1.3e-08)
# Embedded Networks			

Pearson correlation coefficients, p-values are presented in brackets (2-tailed, uncorrected), ** indicate $p \leq 0.0001$, * indicate $p < 0.005$. Source data are provided as a Source Data file. DS = Discovery Sample, RS1 = Replication Sample 1, RS2 = Replication Sample 2, ASD = Autism Sample

Table S2. Behavioral performance in the why/how task (fMRI)

	Social Inferences (why)		Factual Inferences (how)	
	Faces	Hands	Faces	Hands
DS				
Accuracy [%]	93.18 (4.65)	94.29 (5.60)	95.46 (3.21)	95.50 (4.41)
Response time [sec]	0.65 (0.14)	0.71 (0.12)	0.55 (0.11)	0.68 (0.11)
d-prime	3.26 (0.75)	3.55 (0.87)	3.52 (0.67)	3.74 (0.70)
RS1				
Accuracy [%]	90.07 (4.43)	94.88 (5.56)	97.41 (2.79)	93.32 (3.62)
Response time [sec]	0.95 (0.14)	0.98 (0.16)	0.81 (0.13)	0.93 (0.14)
d-prime	2.79 (0.54)	3.47 (0.82)	3.96 (0.67)	3.09 (0.55)
ASD				
Accuracy [%]	88.93 (6.74)	90.63 (7.33)	96.50 (4.33)	91.63 (4.37)
Response time [sec]	1.00 (0.18)	1.04 (0.17)	0.84 (0.15)	0.97 (0.15)
d-prime	2.76 (0.73)	2.88 (0.90)	3.82 (0.86)	2.97 (0.55)
RS2				
Accuracy [%]	89.80 (4.77)	94.96 (4.46)	96.63 (3.68)	96.43 (3.02)
Response time [sec]	0.84 (0.10)	0.86 (0.09)	0.70 (0.08)	0.81 (0.09)
d-prime	2.72 (0.53)	3.51 (0.78)	3.82 (0.76)	3.69 (0.66)

Mean (± SD); Note that the why/how social inference task performed by the discovery sample (DS) differed in several details from the task version performed by the healthy replication sample (RS) and the ASD group. Source data are provided as a Source Data file.

Table S3. Block-specific question endings used in the why/how task.

	Stimulus category		
	Faces	Hands	Non-Social
DS			
WHY (social inference)			
	Is the person…	Is the person…	
	…admiring someone?	…competing against others?	-
	…expressing self-doubt?	…concerned with their health?	-
	…in an argument?	…helping someone?	-
	…proud of themselves?	…protecting themselves?	-
HOW (factual)			
	…looking at the camera?	…lifting something?	-
	…looking to their side?	…pressing a button?	-
	…opening their mouth?	…reaching for something?	-
	…smiling?	…using both hands?	-
RS1, RS2, and ASD			
WHY (social inference)			
	Is the person…	Is the person…	Is it a result of…
	…being affectionate?	…protecting themselves?	…Spring season?
	…expressing self-doubt?	…helping someone?	…a drought?
	…proud of themselves?	…doing their job?	…a forest fire?
	…celebrating something?	…competing against others?	…a hurricane?
	…expressing gratitude?	…expressing themselves?	…a rainstorm?
	…in an argument?	…sharing knowledge?	…going to result in a rainstorm?
HOW (factual)			
	Is the person…	Is the person…	Is the photo showing…
	…looking at the camera?	…carrying something?	…clouds?
	…showing their teeth?	…lifting something up?	…colorful flowers?
	…gazing up?	…putting something on?	…dry ground?
	…opening their mouth?	…reaching for something?	…moving water?
	…looking to the side?	…using a writing utensil?	…palm trees?
	…smiling?	…using both hands?	…smoke?

Table S4. Brain regions decoding social inferences in Face blocks in the why/how fMRI task in the Discovery Sample (DS)

Brain region	Side	k	t	MNI		
				x	y	z
pSTG/SMG (supramarginal gyrus)	L	778	7.50	-56	-48	38
pSTG	R	83	6.31	48	-60	20
SMG	R	471	6.79	50	-44	42
DMPFC	L	493	9.59	-8	56	32
DMPFC/SMA	L	9	5.83	-8	32	52
DMPFC	R	284	7.19	6	52	28
DLPFC	L	331	8.94	-48	14	30
DLPFC	R	243	8.04	52	14	20
VLPFC	L	190	7.40	-48	32	8
SFS	L	34	5.62	-30	28	44
SFS	R	7	5.46	30	32	44

Results are reported at a statistical threshold of $p < 0.05$, FWE corrected at voxel level (cluster threshold of 5 voxels); only peak activations of clusters are reported; L = left hemisphere, R = right hemisphere, MNI = Montreal Neurological Institute, k = cluster size in voxels. Simple t-test of participant-specific decoding accuracy maps at the group level as implemented in SPM12.

Table S5. Brain regions decoding social inferences in Hand blocks in the why/how task in the Discovery Sample (DS)

Brain region	Side	k	t	MNI		
				x	y	z
pSTG (posterior superior temporal gyrus)	L	5077	19.09	-46	-66	24
pSTG	R	536	8.93	48	-62	22
MTG (middle temporal sulcus)	L	168	6.53	-62	-14	-12
MTG	L	37	6.14	-54	6	-22
MTG	L	22	5.67	-44	12	-34
DMPFC, extending to DLPFC, VLPFC and SFS	L	2479	10.71	-30	16	52
DMPFC	R	166	7.60	4	58	24
mOFC	L	112	6.46	-8	56	-16
mOFC	R	45	5.73	2	54	-20
PCC/Precuneus	L/R	538	7.11	-6	-52	42
Cerebellum	L	680	10.33	26	-76	-40

Results are reported at a statistical threshold of $p < 0.05$, FWE corrected at voxel level (cluster threshold of 5 voxels); only peak activations of clusters are reported; L = left hemisphere, R = right hemisphere, MNI = Montreal Neurological Institute, k = cluster size in voxels. Simple t-test of participant-specific decoding accuracy maps at the group level as implemented in SPM12.

Table S6. Univariate results of social inference [why vs how] in the Discovery Sample (DS)

Brain region	Side	k	t		MNI	
				x	y	z
[why > how]						
pSTS	L	267	15.29	-48	-66	34
pSTS	R	25	8.68	54	-64	30
MPFC	L	4750	15.15	-10	44	46
Middle frontal gyrus	L	63	9.93	-42	18	48
Inferior frontal gyrus	R	25	6.64	60	28	22
VLPFC	R	82	6.96	38	30	-16
VLPFC to temporal pole and middle temporal gyrus	L	2729	14.14	-60	-8	-22
Temporal pole (to inferior temporal gyrus)	R	688	9.82	52	10	-34
Middle temporal gyrus	L	100	8.39	-48	-34	-2
PCC and Precuneus	L	779	13.98	-8	-44	34
Hippocampus	L	18	7.19	-24	-20	-16
Cerebellum	R	581	11.7	26	-82	-40
Cerebellum	R	63	8.13	4	-56	-48
Cerebellum	L	108	7.67	-26	-78	-36
[how > why]						
SMG (supramarginal gyus, to inferior parietal lobe)	L	2577	12.39	-40	-40	42
SMG	R	1869	9.85	64	-30	36
ITG (inferior temporal gyrus)	L	155	12.79	-52	-60	-2
ITG	R	110	9.51	52	-56	-6
Pre-SMA	L	185	8.62	-26	-2	50
Pre-SMA	R	109	6.44	26	-2	50
LPFC	L	94	8.27	-48	4	24
LPFC	R	30	6.38	48	10	16
Cerebellum	L	19	7.26	-16	-70	-44

Results are reported at a statistical threshold of $p < 0.05$, FWE corrected at voxel level (cluster threshold of 5 voxels) for the conjunction of face and hand blocks (inclusive masking function, SPM12); only peak activations of clusters are reported; L = left hemisphere, R = right hemisphere, MNI = Montreal Neurological Institute, k = cluster size in voxels. Simple t-test at the group level as implemented in SPM12.

Table S7. Shared neural code for social inferences [why vs. how] in the why/how task for ASD and RS1 (cross-sample decoding).

ROI	Decoding Accuracy [%]	P_{adj}
L_pSTS	62 [47, 53] *	0
R_pSTS	55 [47, 52] *	0.03
L_DMPFC	60 [47, 53] *	0
R_DMPFC	60 [48, 52] *	0
L_SFS	60 [48, 52] *	0.001
L_DLPFC	61 [48, 53] *	0
L_VLPPFC	62 [47, 53] *	0.005

* P_{adj} represents FDR corrected p-values in a permutation test (corrected across all 7 ROIs); activation patterns in each ROI obtained for neurotypical individuals (RS1 = Replication Sample 1) allowed decoding social inferences in ASD (Autism Sample), and vice versa.

Table S8. Activation patterns in each brain region of interest (ROI) decoded social inferences

ROI	DS	RS1	RS2	ASD
L_DLPFC'	1.0e-39 * 0.0000	1.0e-13 * 0.0001	1.0e-37 * 0.0000	1.0e-15 * 0.0000
L_DMPFC'	1.0e-39 * 0.0002	1.0e-13 * 0.0149	1.0e-37 * 0.0001	1.0e-15 * 0.0000
L_VLPFC'	1.0e-39 * 0.0861	1.0e-13 * 0.0314	1.0e-37 * 0.1399	1.0e-15 * 0.0003
L_SFS'	1.0e-39 * 0.2443	1.0e-13 * 0.0054	1.0e-37 * 0.0040	1.0e-15 * 0.0000
L_pSTS'	1.0e-39 * 0.0000	1.0e-13 * 0.0000	1.0e-37 * 0.0000	1.0e-15 * 0.0000
R_DMPFC'	1.0e-39 * 0.0148	1.0e-13 * 0.0132	1.0e-37 * 0.0180	1.0e-15 * 0.0246
R_pSTS'	1.0e-39 * 0.0067	1.0e-13 * 0.8107	1.0e-37 * 0.0000	1.0e-15 * 0.1477

Exact p-values, see Figure 1C; ROI = region of interest; DS = Discovery Sample, RS1 = Replication Sample 1, RS2 = Replication Sample 2, ASD = Autism Spectrum Disorder Sample, R_pSTS/L_pSTS = right and left posterior superior temporal sulcus, L_DMPFC/R_DMPFC = right and left dorsomedial prefrontal cortex, L_SFS = left superior frontal sulcus, L_DLPFC = left dorsal lateral prefrontal cortex, L_VLPFC = left ventral lateral prefrontal cortex; values represent p-values of ROI-wise tests against chance level using a one-sample t-test as implemented in MATLAB R2022b. Non-parametric permutation tests yield qualitatively similar results.

Figure S1. Overlap of univariate and multivariate analyses of social inferences in the why/how task in the Discovery Sample (DS).

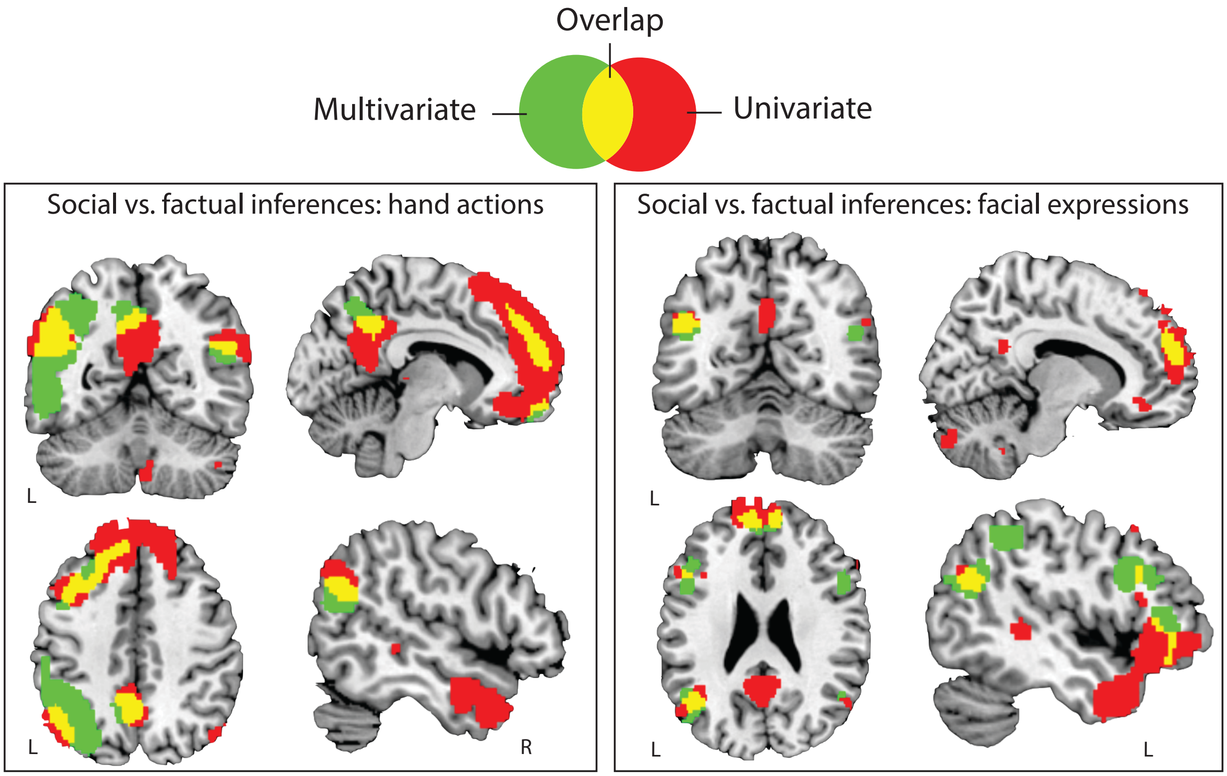

UNIVARIATE RESULTS: For each subject (discovery sample, DS), we contrasted neural responses obtained during [why > how] inferences using block-wise regressors estimated in GLM1, separately for each stimulus set in the why/how task (faces, hands). Individual contrast images were used in two group-level analyses (simple t-tests against baseline as implemented in SPM12). The MULTIVARIATE ANALYSIS of why vs. how task blocks (GLM1) were realized for each subject using a leave-two-block-out cross-validation approach (one why block, one how block in each fold), separately for each stimulus set. Individuals' accuracy maps were then used in a group-level analyses, separately for each stimulus condition (simple t-tests against implicit chance level of 50% for binary classification, as implemented in SPM12). Results of both analyses are displayed at a statistical threshold of $p < 0.05$

FWE corrected at the voxel level for the whole brain, cluster threshold of 5 voxels (separate simple tests as implemented in SPM12).

Figure S2. Correspondence of our pSTS ROI with previous pSTS findings.

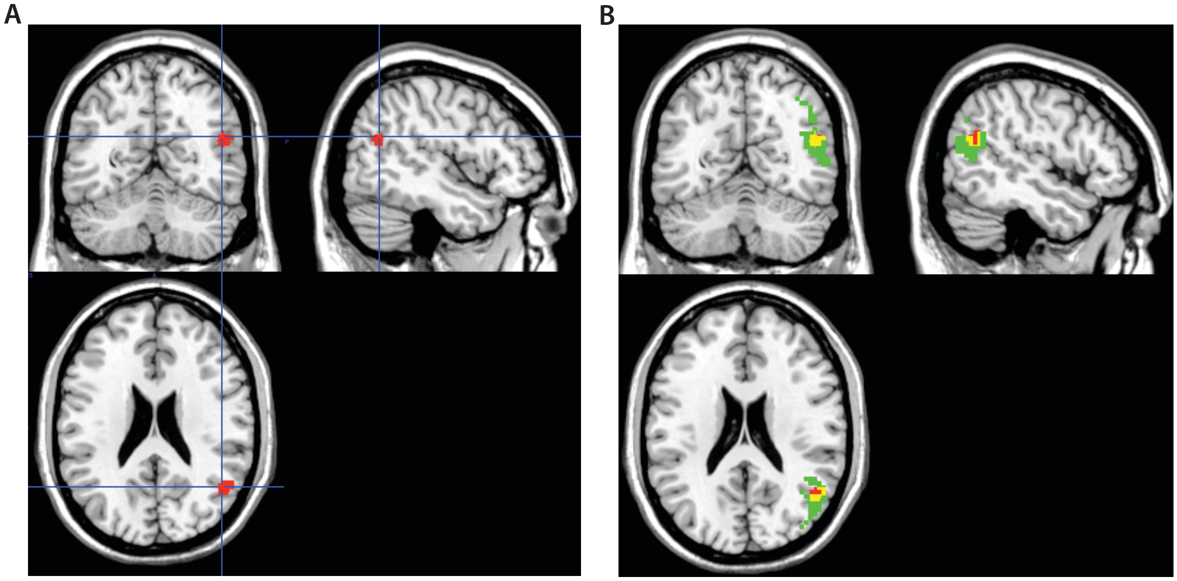

A. Red visualizes the right posterior superior temporal sulcus (pSTS) region of interest (ROI) identified in our why/how task. The blue crosshair illustrates the peak coordinate (MNI space [47.4, -58.8, 22.9]) of responses in the theory of mind (TOM) task in the right hemisphere identified in [14]. B. Red illustrates the right pSTS ROI in our why/how task; green displays the cluster in the pSTS that encoded monetary benefits for other people in an altruistic choice task. Predictive neural information in this green cluster increased when people engaged in theory of mind ("think about how the other person will feel when confronted with your choice") in [15]. Yellow illustrates the overlap of both clusters.

Results S1. Stimulus-specific decoding of social inferences in the why/how task in the Discovery Sample (DS).

Are there brain regions that encoded social inferences significantly more – or less – depending on the stimulus set (hands/faces) in the why/how task? To address this question, we compared whole-brain decoding accuracy maps of social inferences obtained for face blocks and hand blocks of the why/how task (see Table S4 and Table S5 for details on predictive cluster obtained for each stimulus set). Formal comparisons of decoding maps for social inferences obtained in [Faces > Hands] did not yield significant results ($p < 0.05$ FWE corrected at the voxel level, cluster threshold $k = 5$ voxels; paired t-test as implemented in SPM12). The reverse contrast [Hands > Faces] identified a cluster in the left pSTS/ TPJ ([MNI -38, -66, 40], $t = 6.50$, 936 voxels) and right Cerebellum ([MNI 28, -78, -40], $t = 5.58$, 119 voxels), suggesting that images of intentional hand actions more effectively elicited social inferences in both areas (reflected in higher decoding accuracies). Importantly, the identified cluster in the pSTS/TPJ overlapped with the pSTS cluster described in Table 2. Overall, these supplemental results demonstrate a close match of recruited brain areas and predictive information across stimulus categories. The findings also suggest that our data-driven approach of defining ROIs did not systematically exclude brain areas selectively recruited for one but not the other stimulus set in the why/how task.

Methods S1. Detailed description of fMRI data preprocessing in RS2 (fmriPrep)

Results included in this manuscript come from preprocessing performed using *fMRIPrep* 1.5.3 [1], which is based on *Nipype* 1.3.1 [2]. RS2 = Replication Sample 2.

Anatomical data preprocessing

A total of 6 T1-weighted (T1w) images were found within the input BIDS dataset. All of them were corrected for intensity non-uniformity (INU) with N4BiasFieldCorrection [3], distributed with ANTs 2.2.0 [4]. The T1w-reference was then skull-stripped with a *Nipype* implementation of the antsBrainExtraction.sh workflow (from ANTs), using OASIS30ANTs as target template. Brain tissue segmentation of cerebrospinal fluid (CSF), white-matter (WM) and gray-matter (GM) was performed on the brain-extracted T1w using fast [5] from FSL 5.0.9. A T1w-reference map was computed after registration of 6 T1w images (after intensity non-uniformity correction) using mri_robust_template from FreeSurfer 6.0.1 [6]. Volume-based spatial normalization to one standard space (MNI152NLin6Asym) was performed through nonlinear registration with antsRegistration (ANTs 2.2.0), using brain-extracted versions of both T1w reference and the T1w template. The following template was selected for spatial normalization: *FSL's MNI ICBM 152 non-linear 6th Generation Asymmetric Average Brain Stereotaxic Registration Model* [RRID:SCR_002823; TemplateFlow ID: MNI152NLin6Asym].

Functional data preprocessing

For each of the BOLD runs found per subject (across all tasks and sessions), the following preprocessing was performed. First, a reference volume and its skull-stripped version were generated using a custom

methodology of *fMRIPrep*. A B0-nonuniformity map (or *fieldmap*) was estimated based on two (or more) echo-planar imaging (EPI) references with opposing phase-encoding directions, with 3dQwarp from AFNI 20160207 [7]. Based on the estimated susceptibility distortion, a corrected EPI (echo-planar imaging) reference was calculated for a more accurate co-registration with the anatomical reference. The BOLD reference was then co-registered to the T1w reference using flirt [8] from FSL 5.0.9, with the boundary-based registration cost-function [9]. Co-registration was configured with nine degrees of freedom to account for distortions remaining in the BOLD reference. Head-motion parameters with respect to the BOLD reference (transformation matrices, and six corresponding rotation and translation parameters) are estimated before any spatiotemporal filtering using mcflirt [8] from FSL 5.0.9. BOLD runs were slice-time corrected using 3dTshift from AFNI 20160207 [7]. The BOLD time-series (including slice-timing correction when applied) were resampled onto their original, native space by applying a single, composite transform to correct for head-motion and susceptibility distortions. These resampled BOLD time-series will be referred to as *preprocessed BOLD in original space*, or just *preprocessed BOLD*. The BOLD time-series were resampled into standard space, generating a *preprocessed BOLD run in MNI152NLin6Asym space*. First, a reference volume and its skull-stripped version were generated using a custom methodology of *fMRIPrep*. Automatic removal of motion artifacts using independent component analysis (ICA-AROMA) [10] was performed on the *preprocessed BOLD on MNI space* time-series after removal of non-steady state volumes and spatial smoothing with an isotropic, Gaussian kernel of 6mm FWHM (full-width half-maximum). Corresponding "non-aggresively" denoised runs were produced after such smoothing. Additionally, the "aggressive" noise-regressors were collected and placed in the corresponding confounds file. Several confounding time-series were calculated based on the *preprocessed BOLD*: framewise displacement (FD), DVARS and

three region-wise global signals. FD and DVARS are calculated for each functional run, both using their implementations in *Nipype* [following the definitions by [11]. The three global signals are extracted within the CSF, the WM, and the whole-brain masks. Additionally, a set of physiological regressors were extracted to allow for component-based noise correction (*CompCor)* [12]. Principal components are estimated after high-pass filtering the *preprocessed BOLD* time-series (using a discrete cosine filter with 128s cut-off) for the two *CompCor* variants: temporal (tCompCor) and anatomical (aCompCor). tCompCor components are then calculated from the top 5% variable voxels within a mask covering the subcortical regions. This subcortical mask is obtained by heavily eroding the brain mask, which ensures it does not include cortical GM regions. For aCompCor, components are calculated within the intersection of the aforementioned mask and the union of CSF and WM masks calculated in T1w space, after their projection to the native space of each functional run (using the inverse BOLD-to-T1w transformation). Components are also calculated separately within the WM and CSF masks. For each CompCor decomposition, the *k* components with the largest singular values are retained, such that the retained components' time series are sufficient to explain 50 percent of variance across the nuisance mask (CSF, WM, combined, or temporal). The remaining components are dropped from consideration. The head-motion estimates calculated in the correction step were also placed within the corresponding confounds file. The confound time series derived from head motion estimates and global signals were expanded with the inclusion of temporal derivatives and quadratic terms for each [13]. Frames that exceeded a threshold of 0.5 mm FD or 1.5 standardised DVARS were annotated as motion outliers. All resamplings can be performed with *a single interpolation step* by composing all the pertinent transformations (i.e. head-motion transform matrices, susceptibility distortion correction when available, and co-registrations to anatomical and output spaces). Gridded (volumetric)

resamplings were performed using antsApplyTransforms (ANTs), configured with Lanczos interpolation to minimize the smoothing effects of other kernels. Non-gridded (surface) resamplings were performed using mri_vol2surf (FreeSurfer).

Many internal operations of *fMRIPrep* use *Nilearn* 0.6.0 [RRID:SCR_001362], mostly within the functional processing workflow.

Supplementary References

[1] O. Esteban *et al.*, “fMRIPrep: a robust preprocessing pipeline for functional MRI,” *Nat. Methods*, vol. 16, no. 1, pp. 111–116, Jan. 2019, doi: 10.1038/s41592-018-0235-4. [Online]. Available: http://dx.doi.org/10.1038/s41592-018-0235-4

[2] K. Gorgolewski *et al.*, “Nipype: a flexible, lightweight and extensible neuroimaging data processing framework in python,” *Front. Neuroinform.*, vol. 5, p. 13, Aug. 2011, doi: 10.3389/fninf.2011.00013. [Online]. Available: http://dx.doi.org/10.3389/fninf.2011.00013

[3] N. J. Tustison *et al.*, “N4ITK: improved N3 bias correction,” *IEEE Trans. Med. Imaging*, vol. 29, no. 6, pp. 1310–1320, Jun. 2010, doi: 10.1109/TMI.2010.2046908. [Online]. Available: http://dx.doi.org/10.1109/TMI.2010.2046908

[4] B. B. Avants, C. L. Epstein, M. Grossman, and J. C. Gee, “Symmetric diffeomorphic image registration with cross-correlation: evaluating automated labeling of elderly and neurodegenerative brain,” *Med. Image Anal.*, vol. 12, no. 1, pp. 26–41, Feb. 2008, doi: 10.1016/j.media.2007.06.004. [Online]. Available: http://dx.doi.org/10.1016/j.media.2007.06.004

[5] Y. Zhang, M. Brady, and S. Smith, “Segmentation of brain MR images through a hidden Markov random field model and the expectation-maximization algorithm,” *IEEE Trans. Med. Imaging*, vol. 20, no. 1, pp. 45–57, Jan. 2001, doi: 10.1109/42.906424. [Online]. Available: http://dx.doi.org/10.1109/42.906424

[6] B. Fischl, “FreeSurfer,” *Neuroimage*, vol. 62, no. 2, pp. 774–781, Aug. 2012, doi: 10.1016/j.neuroimage.2012.01.021. [Online]. Available: http://dx.doi.org/10.1016/j.neuroimage.2012.01.021

[7] R. W. Cox, "AFNI: what a long strange trip it's been," *Neuroimage*, vol. 62, pp. 743–747, Aug. 2012, doi: 10.1016/j.neuroimage.2011.08.056. [Online]. Available: http://dx.doi.org/10.1016/j.neuroimage.2011.08.056

[8] M. Jenkinson, P. Bannister, M. Brady, and S. Smith, "Improved Optimization for the Robust and Accurate Linear Registration and Motion Correction of Brain Images," *Neuroimage*, vol. 17, no. 2, pp. 825–841, Oct. 2002, doi: 10.1006/nimg.2002.1132. [Online]. Available: http://www.sciencedirect.com/science/article/pii/S1053811902911328

[9] D. N. Greve and B. Fischl, "Accurate and robust brain image alignment using boundary-based registration," *Neuroimage*, vol. 48, pp. 63–72, Oct. 2009, doi: 10.1016/j.neuroimage.2009.06.060. [Online]. Available: http://dx.doi.org/10.1016/j.neuroimage.2009.06.060

[10] R. H. R. Pruim, M. Mennes, D. van Rooij, A. Llera, J. K. Buitelaar, and C. F. Beckmann, "ICA-AROMA: A robust ICA-based strategy for removing motion artifacts from fMRI data," *Neuroimage*, vol. 112, pp. 267–277, May 2015, doi: 10.1016/j.neuroimage.2015.02.064. [Online]. Available: http://dx.doi.org/10.1016/j.neuroimage.2015.02.064

[11] J. D. Power, K. A. Barnes, A. Z. Snyder, B. L. Schlaggar, and S. E. Petersen, "Spurious but systematic correlations in functional connectivity MRI networks arise from subject motion," *Neuroimage*, vol. 59, pp. 2142–2154, Feb. 2012, doi: 10.1016/j.neuroimage.2011.10.018. [Online]. Available: http://dx.doi.org/10.1016/j.neuroimage.2011.10.018

[12] Y. Behzadi, K. Restom, J. Liau, and T. T. Liu, "A component based noise correction method (CompCor) for BOLD and perfusion based fMRI," *Neuroimage*, vol. 37, no. 1, pp. 90–101, Aug. 2007, doi: 10.1016/j.neuroimage.2007.04.042. [Online]. Available: http://dx.doi.org/10.1016/j.neuroimage.2007.04.042

[13] T. D. Satterthwaite *et al.*, “An improved framework for confound regression and filtering for control of motion artifact in the preprocessing of resting-state functional connectivity data,” *Neuroimage*, vol. 64, pp. 240–256, Jan. 2013, doi: 10.1016/j.neuroimage.2012.08.052. [Online]. Available: http://dx.doi.org/10.1016/j.neuroimage.2012.08.052

[14] B. Deen, K. Koldewyn, N. Kanwisher, & R. Saxe (2015). Functional organization of social perception and cognition in the superior temporal sulcus. Cerebral cortex, 25(11), 4596-4609.

[15] A. Tusche & C. A. Hutcherson (2018). Cognitive regulation alters social and dietary choice by changing attribute representations in domain-general and domain-specific brain circuits. Elife, 7, e31185.
