# Supplementary material for: Neural signatures of social inferences predict the number of real-life social contacts and autism severity
Source: Nat Commun. 2023 Jul 20;14:4399. doi: 10.1038/s41467-023-40078-3 (PMC10359299; doi:10.1038/s41467-023-40078-3)
Supplement: Supplementary file 2 — Reporting Summary [file 41467_2023_40078_MOESM2_ESM.pdf]

nature portfolio

Corresponding author(s): Anita Tusche

Last updated by author(s): Jun 7, 2023

Reporting Summary

Nature Portfolio wishes to improve the reproducibility of the work that we publish. This form provides structure for consistency and transparency in reporting. For further information on Nature Portfolio policies, see our Editorial Policies and the Editorial Policy Checklist.

Statistics

For all statistical analyses, confirm that the following items are present in the figure legend, table legend, main text, or Methods section.

n/a	Confirmed	
☐	☒	The exact sample size (*n*) for each experimental group/condition, given as a discrete number and unit of measurement
☐	☒	A statement on whether measurements were taken from distinct samples or whether the same sample was measured repeatedly
☐	☒	The statistical test(s) used AND whether they are one- or two-sided<br>*Only common tests should be described solely by name; describe more complex techniques in the Methods section.*
☒	☐	A description of all covariates tested
☐	☒	A description of any assumptions or corrections, such as tests of normality and adjustment for multiple comparisons
☐	☒	A full description of the statistical parameters including central tendency (e.g. means) or other basic estimates (e.g. regression coefficient) AND variation (e.g. standard deviation) or associated estimates of uncertainty (e.g. confidence intervals)
☐	☒	For null hypothesis testing, the test statistic (e.g. *F*, *t*, *r*) with confidence intervals, effect sizes, degrees of freedom and *P* value noted<br>*Give P values as exact values whenever suitable.*
☒	☐	For Bayesian analysis, information on the choice of priors and Markov chain Monte Carlo settings
☒	☐	For hierarchical and complex designs, identification of the appropriate level for tests and full reporting of outcomes
☐	☒	Estimates of effect sizes (e.g. Cohen's *d*, Pearson's *r*), indicating how they were calculated

Our web collection on statistics for biologists contains articles on many of the points above.

Software and code

Policy information about availability of computer code

Data collection	fMRI Why/How task: stimulus presentation and response recording used the Psychophysics Toolbox (versions 3.0.13 to 3.0.16) operating in MATLAB R2021a (see hyperlinks in the manuscript for code for each task version).
Data analysis	Behavioral and neural data were analyzed using MATLAB R2021a. All fMRI data were analyzed using SPM12 (https://www.fil.ion.ucl.ac.uk/spm/). See the hyperlinks in the manuscript for code regarding preprocessing and first level GLMs. ROI-wise FDR correction was implemented using the fdr_hr function in MATLAB. Code underling the MVPA analysis is openly available (TDT – The Decoding Toolbox, Hebart, M. N., Görgen, K., & Haynes, J. D. (2015). The Decoding Toolbox (TDT): a versatile software package for multivariate analyses of functional imaging data. Frontiers in neuroinformatics, 8, 88) and is based on LIBSVM (a popular library for support vector machines; library available here: https://www.csie.ntu.edu.tw/~cjlin/libsvm/).

For manuscripts utilizing custom algorithms or software that are central to the research but not yet described in published literature, software must be made available to editors and reviewers. We strongly encourage code deposition in a community repository (e.g. GitHub). See the Nature Portfolio guidelines for submitting code & software for further information.

Data

Policy information about availability of data

All manuscripts must include a data availability statement. This statement should provide the following information, where applicable:

- Accession codes, unique identifiers, or web links for publicly available datasets
- A description of any restrictions on data availability
- For clinical datasets or third party data, please ensure that the statement adheres to our policy

The brain data (fMRI) for all neurotypical data generated in this study are available here: Conte Social Inference and Context collection at https://nda.nih.gov/edit_collection.html?id=2643; fMRI data for the autism sample as can be found on the Open Science Framework (OSF, DOI 10.17605/OSF.IO/RNT8S). Source data are provided a de-identified form with this paper and on OSF (DOI 10.17605/OSF.IO/RNT8S).

Human research participants

Policy information about studies involving human research participants and Sex and Gender in Research.

Reporting on sex and gender	Sex at birth was assessed using self-reports and reported for each participant sample (total N: 94 male, 57 female, see Table 1 and de-identified source data file). Sex differences are not hypothesized for the current research question.
Population characteristics	Sociodemographic information (sex, age, IQ) for each participant sample (see Table 1 and in the source data file). All participants in the Autism (ASD) group had a prior clinical diagnosis of autism spectrum disorder, which was confirmed by revised algorithm scores on the Autism Diagnostic Observation Schedule (ADOS), Module 4.
Recruitment	Neurotypical adults (DS, RS1, RS2 groups) were recruited from the Los Angeles area via Craigslist and publicly distributed flyers over the course of the past 8 years. We can't rule out a self-selection bias toward people who are broadly interested in science / psychology / neuroscience. It is also possible that relative to the US population, the sample may have been more racially diverse because we recruited in a major metropolitan area. High-functioning participants with ASD were recruited from our laboratory's registry. All ASD participants met DSM-5/ICD-10 diagnostic criteria for autism spectrum disorder, and all met the cutoff scores for ASD on the Autism Diagnostic Observation Schedule-2 (ADOS-2) revised scoring system for Module 4. All participants completed a version of the why/how fMRI task and a separate behavioral session in exchange for financial compensation ($20/h).
Ethics oversight	Institutional Review Board of the California Institute of Technology ((#12-0343).

Note that full information on the approval of the study protocol must also be provided in the manuscript.

Field-specific reporting

Please select the one below that is the best fit for your research. If you are not sure, read the appropriate sections before making your selection.

☒ Life sciences ☐ Behavioural & social sciences ☐ Ecological, evolutionary & environmental sciences

For a reference copy of the document with all sections, see nature.com/documents/nr-reporting-summary-flat.pdf

Life sciences study design

All studies must disclose on these points even when the disclosure is negative.

Sample size	The discovery sample (DS), replication sample 1 (RS1), and autism sample (ASD) were based on existing data (DS=59, RS1=20, ASD=23, see Table 1), taking advantage of their study design (Why/How task: block design, see Table 5) and sample sizes. We collected a separate replication sample (RS2, n=55, see Table 1) of approximately the same size as DS to replicate the effects found in the DS group. No statistical method was used to predetermine sample size. The replicability of our main effects across four data sets demonstrates that the sample size is sufficient to detect the effects of interest. For the prediction in the smaller participant sample of the ASD group (n=25), we used a cross-sample prediction approach: we trained our model on data from all neurotypical subject samples (n>100) and tested the model on data from the ASD group. This approach addresses potential methodological concerns related to the smaller sample size in the ASD group (i.e., concerns of limited training data in a leave-one-subject-out cross-validation approach in a small ASD sample).
Data exclusions	We excluded data from one participant in the DS group and one participant in the RS2 group due to poor performance in the why/how fMRI task (no responses to > 70% of trials). We excluded data from two individuals in the ASD group due to artifacts in the fMRI data. We excluded data from one participant in RS2 due to a SNI score of 106 that exceeded three standard deviations above the group average (Table 1) and another three individuals in the RS2 group due to excessive motion in the scanner. Motion outliers at the individual level were identified and excluded based on the approach described in references 75,76 (see manuscript), which uses low pass filtering to control false rejections associated with respiration and pseudo-motion signals present in short repetition time multi-band EPI sequences.
Replication	The study contained multiple replications of the original findings in the discovery sample (DS), including two neurotypical replication samples (RS1, RS2) and one autism sample (ASD). For the prediction in the smaller participant sample of the ASD group, we used a cross-sample

	prediction approach: we trained our model on data from all neurotypical subject samples and tested the model on data from the ASD group. This approach addresses potential methodological concerns related to the smaller sample size in the ASD group.
Randomization	All participant samples completed a version of the Why/How fMRI task (Table 5) and a battery of behavior measures to characterize social functioning. Participant samples were recruited over time (to replicate core results and examine generalization to an ASD sample). No allocation to different conditions or experimental manipulations was used; hence, no randomization of allocation was used. That said, block order within the why/how task was pseudo-randomized.
Blinding	All participant samples completed a version of the Why/How fMRI task and a battery of behavior measures to characterize social functioning. As all participants provided the same information and experimental task, no blinding of the investigator to (non-existent) experimental conditions was applied.

Reporting for specific materials, systems and methods

We require information from authors about some types of materials, experimental systems and methods used in many studies. Here, indicate whether each material, system or method listed is relevant to your study. If you are not sure if a list item applies to your research, read the appropriate section before selecting a response.

Materials & experimental systems

n/a	Involved in the study
☒	☐ Antibodies
☒	☐ Eukaryotic cell lines
☒	☐ Palaeontology and archaeology
☒	☐ Animals and other organisms
☒	☐ Clinical data
☒	☐ Dual use research of concern

Methods

n/a	Involved in the study
☒	☐ ChIP-seq
☒	☐ Flow cytometry
☐	☒ MRI-based neuroimaging

Magnetic resonance imaging

Experimental design

Design type	task-based, block design (see Table 5 for hyperlinks to the source code of the fMRI task versions)
Design specifications	Task details varied across versions of the Why/How task. Table 5 provides detailed information about task conditions, blocks per condition, stimuli per block, timing, and source code, among other details. Supplemental Table S3 provides block-specific questions used in each task version.
Behavioral performance measures	Performance in the why/how task was assessed as follows using participants' button presses during the task: for each participant, we computed measures of mean percent accuracy, d' and response time (RT) for the four conditions (inference [why, how] x targets [faces, hands]). Table S2 reports group-level descriptive statistics (mean, standard deviations) for each measure, condition, and participant sample (also see the source data file).

Acquisition

Imaging type(s)	functional
Field strength	3T
Sequence & imaging parameters	All imaging data were acquired at the Caltech Brain Imaging Center. Imaging data of DS, RS1 and ASD were collected using a Siemens Trio 3.0 Tesla MRI scanner outfitted with a 32-channel phased-array head coil. For the DS, we acquired 304 whole-brain T2*-weighted echoplanar image volumes (EPIs; voxel resolution = 2.5 x 2.5 x 2.5 mm3, 56 slices, TR = 1000 ms, TE = 30 ms, flip angle = 60°, FOV = 200 mm, interleaved acquisition order, multi-band acceleration factor = 4) for the why/how social inference task. For the RS1 and ASD groups, whole-brain T2*-weighted EPI volumes for the why/how task were acquired with the following MR protocol: voxel resolution = 3 x 3 x 3 mm3, 47 slices, TR = 2500 ms, TE = 30 ms, flip angle = 85°, FOV = 192 mm, ascending acquisition order. For RS2, imaging data were collected at Siemens 3.0 Tesla MAGNETOM Prisma.Fit MRI scanner outfitted with a 32-channel phased-array head coil. For RS2, we acquired 1080 whole-brain T2*-weighted EPI volumes with the following parameters: TR = 700 ms, TE = 30 ms, 60 contiguous oblique transverse slices, slice pitch 20 degrees, 2.5 mm isotropic voxel size, multi-band acceleration = 6, interleaved slice acquisition order, flip angle = 53 degrees. A pair of spin echo EPI volumes (TR = 5500 ms, TE = 48 ms, and multi-band acceleration = 1) with opposing phase encoding polarity were acquired for geometric distortion correction with identical geometry and EPI echo spacing to the T2*-weighted EPI volumes.
Area of acquisition	whole brain

Diffusion MRI ☐ Used ☒ Not used

Preprocessing

Preprocessing software

SPM 12

Normalization

The group-wise DARTEL registration method included in SPM12 was used to normalize the T1 structural volume to a common group-specific space, with subsequent affine registration to Montreal Neurological Institute (MNI) space. All EPI volumes were normalized to MNI space using the deformation flow fields generated in the previous step, which simultaneously re-sampled volumes to 2 mm isotropic.

Normalization template

MNI space (SPM12)

Noise and artifact removal

Motion outliers at the individual level were identified and excluded based on the approach described in [75,76] (see main manuscript), which uses low pass filtering to control false rejections associated with respiration and pseudo-motion signals present in short repetition time multi-band EPI sequences. The six rigid body motion parameters estimated during pre-preprocessing were low pass filtered temporally using a fifth-order Butterworth filter with a critical frequency of 0.2 Hz. Following filtering, the framewise displacement (FD) time series were calculated for each participant [77] (see main manuscript). Participants with unusually high motion were identified from the set of individual 50th and 95th percentile frame-wise displacement (FD) values using the DBSCAN clustering algorithm [78] (see main manuscript) implemented by scikit-learn [79] (see main manuscript).

Volume censoring

No formal volume censoring was applied beyond the preprocessing steps described above (see noise and artifact removal).

Statistical modeling & inference

Model type and settings

GLM: For each participant, a general linear model (GLM) estimated regressors of interest for each block of the why/how task. Blocks were defined by the onset of the first target image and the offset of the final image of the block [86]. The GLM included as covariates of no interest the six motion parameters estimated from image realignment and a predictor for every time point where in-brain frame-wise signal change (calculated as the root mean square derivate, or DVARS) exceeded 2.5 SDs of the mean DVARS across the time series or where frame-wise displacement exceeded 0.5 mm of translation or 0.5° of rotation). The hemodynamic response was modeled using the canonical (double-gamma) response function and a 1/100 Hz high-pass cutoff filter to eliminate low-frequency drifts in data. GLMs were estimated using the SPM12 RobustWLS toolbox, which implements the robust weighted least-squares estimation algorithm [88]. Estimated responses for the block-wise regressors of interest (i.e., 'why' and 'how' inference task-blocks) were used as inputs for the multivariate classification of 'why' vs. 'how' inferences to identify (DS) or confirm (RS1, RS2, ASD) brain regions that decode social inferences in the why/how task (described below). Moreover, for each participant, we estimated one contrast image based on regressors of interest in conditions that included social stimuli ([why face-blocks AND why hand-blocks] – [how face-blocks AND how hand-blocks]). These subject-specific contrast images were used as inputs for the multivariate regression analyses to predict variance in subjects' number of social contacts (SNI scores) (and other behavioral indices of interest such as AQ, SRS-2 or ADOS SA scores). For each subject, we also estimated one matching contrast image for the nonsocial control condition [why nonsocial – how nonsocial]. Note that this contrast was only estimated for subject samples for which the nonsocial task condition was available (Table 5). These subject-wise contrast images were used as input for the post-hoc multivariate regression analysis that probed the social specificity of our neural predictions.

Effect(s) tested

In a first step, we identified brain regions that are reliably engaged during social inference processing in neurotypical individuals in the DS group. More precisely, this initial analysis aimed to identify multi-voxel activation patterns in the brain that decode social (or factual) inferences in the why/how task (i.e., 'why' vs. 'how' task blocks). To this end, we used a linear support vector machine classifier (libSVM, http://www.csie.ntu.edu.tw/~cjlin/libsvm) in combination with a standard whole-brain searchlight approach. Resulting clusters were defined as regions of interest (ROIs) for the multivariate prediction of individual differences in social network characteristics (see below) for all four participant samples. Focusing on a fixed set of brain areas identified in DS minimizes the risk of circular analysis (i.e., double dipping) and producing false positive results in the remaining three subject samples (RS1, RS2, ASD). Next, we ran a support vector regression (SVR) analysis of an individual's SNI scores (labels) and the subject's ROI-based brain responses in the why/how task (features). We repeated this analyses for other indices of social functioning (e.g., symptom severity in ASD).

Specify type of analysis: ☐ Whole brain ☐ ROI-based ☒ Both

Anatomical location(s): functional ROIs based on whole-brain results in the discovery sample, DS

Statistic type for inference
(See Eklund et al. 2016)

We used SPM12's statistical test tools (http://www.fil.ion.ucl.ac.uk/spm) for the initial whole brain analysis (thresholded at $p < 0.05$, FWE correction at the voxel level for the whole brain volume). For the ROI-based analyses, we used non-parametric permutation tests and FDR correction to control for multiple comparisons.

Correction

FWE for whole brain analysis, FDR for ROI-based analysis

Models & analysis

n/a	Involved in the study
☒	☐ Functional and/or effective connectivity
☒	☐ Graph analysis
☐	☒ Multivariate modeling or predictive analysis

Multivariate modeling and predictive analysis

We ran a support vector regression (SVR) analysis of an individual's SNI scores (labels) and the subject's ROI-based brain responses in the fMRI why/how task (features). ROIs (used for feature selection) were based on results of an independent searchlight decoding analysis ('why' vs. 'how' inferences in the why/how task) on brain data of the discovery sample. For each ROI (Table 2), we performed the following analysis steps: first, for every participant, we extracted parameter estimates for all voxels in an ROI (Figure 1B) from the contrast image of individuals' GLM ([why face-blocks AND why hand-blocks] – [how face-blocks AND how hand-blocks]). The resulting neural pattern vectors (one per participant) were used as input features for the prediction, and the participant's number of social contacts (SNI scores) served as labels. Predictions used a linear v-SVR (libSVM) with a fixed cost parameter c = 1 together with a leave-one-subject-out approach. We then tested if the model could predict the SNI score of the remaining participant solely based on this subject's ROI-specific neural activation pattern ('test data'). ROI-specific prediction accuracies reflect correlations of the observed and predicted social network score across participants in each sample. Permutation tests assessed the statistical significance of the prediction by comparisons to the empirical null distribution estimated for this ROI and sample (realized by randomly permuting the pairing of subjects' neural pattern vectors and behavioral SNI scores 1000 times). Only predictions above the 95th percentile of null distributions that survived FDR correction across the seven ROIs were considered statistically significant for subjects in DS. FDR correction was implemented using the fdr_hr function in MATLAB. These analysis steps were repeated for data from the RS1 and RS2 groups, as well as for other indices of social functioning such as AQ and SRS-2 scores and WASI-II IQ scores. ROIs were identical across all four subject samples to explicitly test the generalizability of our findings in DS to data from other subject samples.
